# Targeting Sirtuins in Thyroid Cancer: Mechanisms, Drug Development, and Emerging Roles in Tumor Immunity and Ferroptosis

**DOI:** 10.3390/cancers18132093

**Published:** 2026-06-27

**Authors:** Ki Ju Cho, Ji Hyun Seo, Hayeong Kwon, Seung-Jun Lee, Young-Sool Hah, Jung Je Park

**Affiliations:** 1 Department of Otorhinolaryngology, Myongji Hospital, Hanyang University Medical Center, 55 Hwasu-ro 14 beon-gil, Goyang 10475, Republic of Korea; genkj0107@gmail.com; 2 Department of Otorhinolaryngology-Head and Neck Surgery, Gyeongsang National University College of Medicine, 79 Gangnam-ro, Jinju 52725, Republic of Korea; hykwon@gnu.ac.kr; 3 Institute of Medical Science, Gyeongsang National University College of Medicine, 816-15 Jinju-daero, Jinju 52727, Republic of Korea; seozee@gnu.ac.kr (J.H.S.); 0789zxc@gnu.ac.kr (S.-J.L.); 4 Department of Pediatrics, Gyeongsang National University College of Medicine, 816-15 Jinju-daero, Jinju 52725, Republic of Korea; 5 Department of Surgery, Gyeongsang National University College of Medicine, 816-15 Jinju-daero, Jinju 52725, Republic of Korea; 6 Biomedical Research Institute, Gyeongsang National University Hospital, 79 Gangnam-ro, Jinju 52727, Republic of Korea

**Keywords:** thyroid cancer, sirtuin, SIRT6, SIRT7, desuccinylation, ferroptosis, tumor immunity, *BRAF* V600E, radioiodine refractoriness, anaplastic thyroid cancer

## Abstract

Thyroid cancer (TC) is the most common endocrine malignancy, with incidence increasing worldwide. Although most differentiated TCs have a favorable prognosis, radioiodine (RAI)-refractory differentiated thyroid cancer (DTC), BRAF inhibitor-resistant papillary thyroid cancer, and anaplastic thyroid cancer (ATC) remain major areas of unmet clinical need. The sirtuin (SIRT) family of NAD^+^-dependent enzymes has emerged as a multifaceted regulator of TC biology, with isoform-specific dichotomous roles: SIRT1, SIRT6, and SIRT7 act as tumor promoters through engagement of BRAF/MAPK, PI3K/AKT, epithelial–mesenchymal transition (EMT), and Hippo pathways, while SIRT3 and SIRT4 function as tumor suppressors via mitochondrial metabolic regulation. This review synthesizes recent developments that expand the therapeutic landscape: (i) the recognition that SIRT7 functions as a desuccinylase with preclinically identified oncogenic substrates, modifying KIF23 in ATC and LATS1 in PTC; (ii) the emerging roles of isoform-specific SIRT axes, including the NAMPT–SIRT1–PD-L1 axis, SIRT6-associated regulatory T-cell biology, and SIRT2 as a T-cell metabolic checkpoint, as determinants of immune microenvironment state and potential modulators of immune checkpoint inhibitor response; and (iii) the SIRT6–nuclear receptor coactivator 4 (NCOA4) ferritinophagy axis as a supported ferroptosis vulnerability in ATC, with potential but still hypothesis-generating relevance to dedifferentiated and RAI-refractory DTC. Importantly, the therapeutic logic for SIRT6 is disease-state-specific rather than contradictory: SIRT6 inhibition is rationalized in BRAF-driven aggressive PTC and DTC contexts where SIRT6 supports MAPK signaling, EMT, and ferroptosis resistance, whereas in SIRT6-high ATC, the same enzyme’s NCOA4-dependent ferritinophagy activity may instead be exploited to enhance ferroptosis sensitivity. We review the current SIRT modulator pharmacological toolkit—including EX-527, OSS_128167, and emerging SIRT7-selective inhibitors—and identify the substantial clinical translation gap, with no SIRT-targeted clinical trial yet conducted in TC, despite strong preclinical rationale. We outline biomarker-stratified combination strategies with BRAF/MEK inhibitors, multikinase inhibitors, immune checkpoint inhibitors, and ferroptosis inducers, prioritizing biomarker-driven preclinical validation and, where supported by efficacy and safety data, subsequent early-phase evaluation in *BRAF* V600E-mutant and SIRT6-high thyroid cancer. Sirtuins thus represent a mechanistically promising and potentially biomarker-stratifiable therapeutic hypothesis for difficult-to-treat thyroid cancer; however, clinical translation remains at an early stage and requires validated biomarkers, isoform-selective compounds, and disease-specific in vivo evidence.

## Simple Summary

Thyroid cancer is the most common endocrine malignancy and is increasing rapidly worldwide. While most thyroid cancers can be treated successfully, a substantial group of patients faces poor outcomes when their tumors become refractory to radioactive iodine, when resistance develops to targeted agents, or when they develop the most aggressive form known as anaplastic thyroid cancer. New treatment options are urgently needed for these patients. In this review, we examine a family of seven proteins, the sirtuins, which have emerged as promising drug targets. We summarize how individual sirtuins either drive tumor growth or protect against it, and we describe three recent breakthroughs that connect sirtuins to immune system regulation and to a newly recognized form of cell death. We propose specific drug combinations pairing sirtuin-targeting compounds with existing thyroid cancer therapies, providing a framework that may inform future preclinical studies and, where appropriate, carefully selected early clinical evaluation.

## 1. Introduction

Thyroid cancer (TC) is the most common endocrine malignancy and one of the fastest-growing cancers worldwide. According to the most recent GLOBOCAN 2022 estimates, an estimated 821,214 new TC cases and 47,507 TC-related deaths occurred globally in 2022, with age-standardized incidence rates (ASIR) of 13.60 per 100,000 in women and 4.60 per 100,000 in men, and corresponding age-standardized mortality rates (ASMR) of 0.53 and 0.35 per 100,000, respectively [1]. The Global Burden of Disease (GBD) 2021 study further documented an approximately 185% increase in global TC incidence between 1990 and 2021 [2], with an estimated 2 million prevalent cases as of 2021 [3]. The disease is approximately threefold more common in women than in men and shows substantial geographic variation in incidence [4,5], with the highest ASIRs observed in East Asia and Polynesia (~20–40 per 100,000) and the lowest in much of sub-Saharan Africa (<2 per 100,000) [1,4,5]. Incidence also varies substantially across the Human Development Index (HDI) gradient—very-high HDI countries show ASIRs of approximately 16 per 100,000 in women versus <3 per 100,000 in low HDI countries—reflecting both biological factors (iodine status, body mass index, hormonal exposures) and differences in screening intensity. Notably, the mortality-to-incidence ratio (MIR) has remained low and stable or has declined globally despite rising incidence—a pattern most pronounced for papillary histology and most often attributed to overdiagnosis of indolent small papillary lesions in screening-intensive settings [1,5]. Approximately 64.6% of new TC cases occur in individuals under 55 years of age, contributing substantially to years of life lived with disease and to the long-term clinical burden of surveillance and follow-up care [1]. Histologically, TC encompasses four major subtypes with markedly different clinical behavior: papillary thyroid cancer (PTC, ~85% of cases, 5-year survival > 98%), follicular thyroid cancer (FTC, ~10%, 5-year survival ~92%), medullary thyroid cancer (MTC, ~3–5%), and anaplastic thyroid cancer (ATC, <2%, 5-year survival 5–10%) [6,7]. Despite the overall favorable prognosis of differentiated TC, the disease imposes a substantial clinical burden through three persistent unmet needs: radioiodine (RAI)-refractory differentiated TC (15–20% of advanced cases) [8], BRAF inhibitor-resistant aggressive PTC [9,10], and ATC, which remains one of the most lethal human malignancies (median overall survival of approximately 5–6 months from diagnosis, with 1-year survival below 20%) [7,11].

Current targeted therapy for advanced TC, while improved, remains inadequate. The multikinase inhibitors lenvatinib and sorafenib are standard for RAI-refractory differentiated TC but provide median progression-free survival of only 12–18 months and are limited by substantial toxicity (hypertension, fatigue, mucositis, hand–foot syndrome) [12,13]. For *BRAF* V600E-mutant ATC and PTC, the combination of dabrafenib + trametinib has produced unprecedented response rates but is similarly limited by acquired resistance through MAPK reactivation, HER3 upregulation, and parallel pathway adaptation [9,10]. Cabozantinib has been approved for DTC after lenvatinib failure but does not fundamentally alter the trajectory of refractory disease [14]. Immune checkpoint inhibitors (ICIs)—pembrolizumab, spartalizumab, and nivolumab—produce modest response rates (<30%) in TC, with greater activity in ATC than in advanced PTC due to differences in tumor mutational burden and immune microenvironment [15,16]. Across all these modalities, novel therapeutic approaches are urgently needed, particularly approaches that integrate with rather than replace established backbones.

The sirtuin (SIRT) family of NAD^+^-dependent enzymes has emerged as a particularly promising therapeutic target in TC [17,18]. The seven mammalian sirtuins (SIRT1–SIRT7) regulate diverse biological processes, including transcription, DNA repair, cell cycle progression, metabolic homeostasis, and oxidative stress response, and exhibit isoform-specific roles in cancer that range from tumor promotion to tumor suppression, depending on the cellular and disease context [17,19]. The dichotomous behavior of sirtuins reflects their distinct subcellular localizations (nuclear: SIRT1, SIRT6, SIRT7; cytoplasmic: SIRT2; mitochondrial: SIRT3, SIRT4, SIRT5), substrate preferences, and enzymatic activities—including not only canonical deacetylation but also non-canonical desuccinylation, demalonylation, and ADP-ribosylation [20,21,22]. In TC specifically, SIRT1, SIRT6, and SIRT7 predominantly drive tumor progression by engaging BRAF/MAPK, PI3K/AKT, EMT, and Hippo pathways [23,24,25,26,27,28], while SIRT3 and SIRT4 act as tumor suppressors by regulating mitochondrial metabolism and redox balance [29,30]. SIRT2 and SIRT5 remain underexplored in TC and represent open research questions. This dichotomous role framework provides a unifying conceptual basis for sirtuin-targeted therapy in TC and underpins the structure of the present review.

This review distinguishes itself from prior literature on sirtuins in cancer by integrating recent work published since 2024, which has fundamentally expanded the therapeutic landscape. First, the recognition that SIRT7 functions as a functionally relevant desuccinylase in TC, with kinesin family member 23 (KIF23) in ATC and large tumor suppressor 1 (LATS1) in PTC as the principal oncogenic substrates, has reshaped understanding of post-translational modification regulation in this disease and identified an entirely new class of therapeutic targets [25,27]. Second, integrative reviews of sirtuins in tumor immunity have positioned isoform-specific SIRT axes (NAMPT-SIRT1-PD-L1, SIRT6-Treg, SIRT2-CD8^+^ effector function) as determinants of the immune microenvironment, providing a mechanistic basis for SIRT modulator combinations with immune checkpoint inhibitors [31,32]. Third, the SIRT6–nuclear receptor coactivator 4 (NCOA4) ferritinophagy axis has direct preclinical support as a ferroptosis vulnerability in ATC, whereas its relevance to dedifferentiated and RAI-refractory DTC remains a testable hypothesis [33,34,35]. This distinction is important for designing combination strategies with sulfasalazine, glutathione peroxidase 4 (GPX4) inhibitors, and other ferroptosis-inducing agents. Together, these three trends transform sirtuins from a primarily mechanistic curiosity into a preclinically supported, potentially actionable therapeutic hypothesis warranting further validation. The 2025 American Thyroid Association guidelines, with their emphasis on risk-adapted treatment intensity, provide a framework for integrating SIRT-based patient stratification [36]. The take-home message of this review is that sirtuins represent a mechanistically promising and potentially biomarker-stratifiable therapeutic hypothesis for the most challenging contexts in thyroid cancer therapy, although clinical validation remains at an early stage. Notably, the SIRT framework developed here primarily applies to follicular-derived thyroid cancers (PTC, FTC, ATC); medullary thyroid cancer (MTC), which arises from parafollicular C-cells and is driven by RET-activating mutations, is discussed only briefly and should not be interpreted within the same mechanistic axis (see Section 3.4).

*Aim and central hypothesis.* The central hypothesis of this review is that the sirtuin family constitutes a mechanistically coherent and biomarker-stratifiable therapeutic target class for the most difficult-to-treat contexts in thyroid cancer—BRAF inhibitor-resistant papillary thyroid cancer, radioiodine (RAI)-refractory differentiated thyroid cancer, and anaplastic thyroid cancer—when sirtuin modulators are deployed in an isoform- and disease-state-specific manner and combined with established therapeutic backbones. To examine this hypothesis, we pursue four specific aims: (i) to establish the structural and enzymatic basis for isoform-specific sirtuin function, including the recently recognized non-canonical desuccinylation activity; (ii) to synthesize the dichotomous tumor-promoting (SIRT1, SIRT6, SIRT7) and tumor-suppressive (SIRT3, SIRT4) roles of sirtuins across thyroid cancer subtypes, integrating original transcriptomic analysis with the published literature and explicitly stratifying the level of supporting evidence; (iii) to evaluate the emerging roles of sirtuins in tumor immunity and ferroptosis as determinants of immune checkpoint inhibitor response and ferroptosis-based vulnerability, respectively; and (iv) to translate these mechanistic insights into a biomarker-guided combination-therapy framework and to define the preclinical validation and trial-design priorities required to test it. Throughout, we distinguish thyroid cancer-specific evidence from extrapolation from other cancers, so that the strength of each element of the hypothesis is transparent to the reader.

*Search strategy and scope.* This is a narrative review with a transparent and reproducible search strategy. We did not use a PRISMA-level systematic review framework because the heterogeneity of the underlying evidence (mechanistic cell-line studies, animal models, single-arm clinical trials, mixed-cancer reviews) is not amenable to meta-analytic synthesis, and a critical narrative approach was judged most appropriate for integrating these disparate evidence streams. Literature was retrieved from four databases (PubMed, Scopus, Web of Science, and the Cochrane Library) and supplemented with a targeted search of ClinicalTrials.gov for ongoing or completed trials of SIRT modulators in solid tumors with thyroid cancer relevance. Search terms combined sirtuin-related keywords (“sirtuin,” “SIRT1,” “SIRT2,” “SIRT3,” “SIRT4,” “SIRT5,” “SIRT6,” “SIRT7,” “NAD-dependent deacetylase,” “desuccinylation,” “NAMPT”) with thyroid cancer-related keywords (“thyroid cancer,” “papillary thyroid cancer,” “follicular thyroid cancer,” “anaplastic thyroid cancer,” “medullary thyroid cancer,” “radioiodine-refractory (RAI-refractory),” “*BRAF* V600E”) and mechanism/therapy keywords (“ferroptosis,” “tumor immunity,” “PD-L1,” “immune checkpoint inhibitor,” “drug development,” “combination therapy”). Publication dates ranged from January 2000 to January 2026, and the final search was completed on 31 January 2026. Inclusion criteria were peer-reviewed primary research, mechanistic studies, clinical trials, and high-quality narrative or systematic reviews relevant to sirtuin biology in cancer, with priority for thyroid cancer-specific evidence. Preclinical studies (cell lines, xenografts, organoids) and clinical evidence (case series, phase I/II/III trials, real-world data) are explicitly distinguished throughout the text. Conference abstracts, non-peer-reviewed preprints (except where explicitly indicated), and non-English publications without translated abstracts were excluded. Where thyroid cancer-specific data were unavailable, mechanistic inferences from other cancer types are clearly labeled as such. Of approximately 4060 records initially identified across all sources, 196 references were ultimately included in the review after duplicate removal, title/abstract screening, and full-text assessment. Reference selection emphasized recent literature from 2024 onward, where it represented field-changing developments, complemented by foundational papers establishing sirtuin biology.

## 2. Sirtuin Family: Structure, Localization, and Diverse Enzymatic Activities

**Figure 1:**
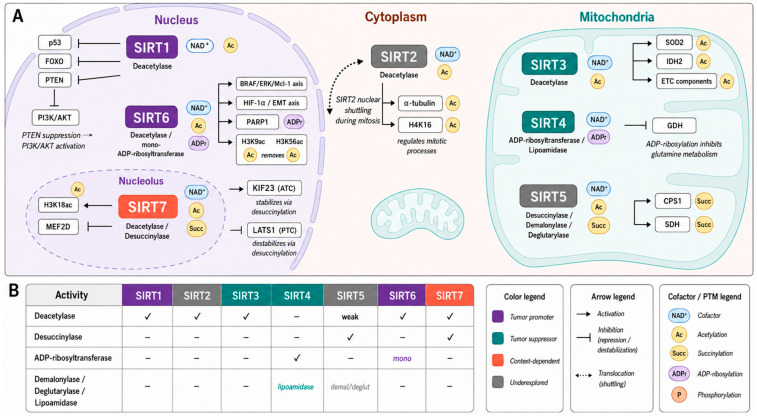
Sirtuin family subcellular localization and enzymatic activities in thyroid cancer. (**A**) The seven mammalian sirtuins (SIRT1–7) display distinct subcellular localizations and enzymatic activities. Nuclear sirtuins SIRT1 and SIRT6 (purple), and SIRT7 (coral, enriched in the nucleolus), predominantly act as tumor promoters or context-dependent regulators in thyroid cancer. SIRT1 inhibits p53, FOXO, and PTEN, thereby activating PI3K/AKT signaling. SIRT6 activates the BRAF/ERK/Mcl-1 and HIF-1α/EMT axes, mono-ADP-ribosylates PARP1, and deacetylates H3K9 and H3K56. SIRT7 functions as both a deacetylase (H3K18, MEF2D) and a desuccinylase, stabilizing KIF23 in anaplastic thyroid cancer and destabilizing LATS1 in papillary thyroid cancer. SIRT2 (grey) is primarily cytoplasmic, deacetylates α-tubulin and H4K16 to regulate mitotic processes, and shuttles to the nucleus during mitosis. Mitochondrial sirtuins SIRT3 and SIRT4 (teal) act as tumor suppressors: SIRT3 activates SOD2, IDH2, and components of the electron transport chain, while SIRT4 ADP-ribosylates and inhibits GDH, suppressing glutamine metabolism in papillary thyroid cancer. SIRT5 (grey) functions as a desuccinylase/demalonylase/deglutarylase regulating CPS1 and SDH; its role in thyroid cancer remains underexplored. (**B**) Summary of sirtuin enzymatic activity diversification across the seven family members. Non-canonical desuccinylation by SIRT5 and SIRT7 represents an emerging paradigm of post-translational modification (PTM) regulation in thyroid cancer (2024–2025). ATC, anaplastic thyroid cancer; CPS1, carbamoyl phosphate synthetase 1; DTC, differentiated thyroid cancer; EMT, epithelial–mesenchymal transition; ETC, electron transport chain; FTC, follicular thyroid cancer; GDH, glutamate dehydrogenase; IDH2, isocitrate dehydrogenase 2; KIF23, kinesin family member 23; LATS1, large tumor suppressor 1; MEF2D, myocyte enhancer factor 2D; PARP1, poly(ADP-ribose) polymerase 1; PTC, papillary thyroid cancer; SDH, succinate dehydrogenase; SOD2, superoxide dismutase 2.

The mammalian sirtuin family comprises seven members (SIRT1–SIRT7) that share a conserved catalytic core of approximately 275 amino acids organized into a small zinc-binding domain and a larger Rossmann-fold characteristic of NAD^+^-binding proteins [37,38,39]. Despite this structural conservation, the seven sirtuins differ markedly in their N- and C-terminal extensions, subcellular localization, substrate preferences, and enzymatic activities—a diversity that underlies their pleiotropic and often opposing biological functions in thyroid cancer (Section 3 and Section 4) [17,18]. Figure 1 summarizes these features schematically. This section provides the foundational framework for interpreting the dichotomous roles developed in subsequent sections, with particular emphasis on the non-canonical enzymatic activities that have reshaped sirtuin biology since 2024 [22,25,27].

### 2.1. Classification and Subcellular Localization

The seven mammalian sirtuins are distributed across three major subcellular compartments. SIRT1, SIRT6, and SIRT7 are predominantly nuclear, with SIRT1 also undergoing cytoplasmic shuttling under stress conditions and SIRT7 specifically enriched in the nucleolus, where it regulates ribosomal biogenesis [40,41]. SIRT2 is the principal cytoplasmic sirtuin and translocates to the nucleus during specific cell cycle phases to regulate mitotic processes [42,43]. SIRT3, SIRT4, and SIRT5 reside in the mitochondrial matrix, where they function as key regulators of mitochondrial protein acylation and metabolic enzyme activity [29,44,45,46]. This compartmentalization is functionally meaningful: the nuclear sirtuins predominantly engage histone, transcription factor, and DNA repair substrates, while the mitochondrial sirtuins target metabolic enzymes and components of the electron transport chain [19,47]. SIRT2’s cytoplasmic localization aligns with its role in cytoskeletal regulation through tubulin deacetylation [42,48].

### 2.2. Canonical Deacetylase Activity

The canonical sirtuin reaction is the NAD^+^-dependent deacetylation of acetyl-lysine residues on histone and non-histone substrates, releasing nicotinamide and 2′-O-acetyl-ADP-ribose as products [49,50]. SIRT1, SIRT2, SIRT3, SIRT6, and SIRT7 all possess deacetylase activity, although their substrate preferences differ substantially. SIRT1 deacetylates a broad repertoire, including p53 (K382) [51,52], FOXO transcription factors [53], NF-κB p65 [54], PGC-1α [55], and HIF-1α [56], supporting its role as a central integrator of stress and metabolic signaling. SIRT2 primarily deacetylates α-tubulin K40 and histone H4K16, with additional substrates including FOXO3a and various mitotic regulators [42,43,57]. SIRT3 deacetylates mitochondrial enzymes, including superoxide dismutase 2 (SOD2), isocitrate dehydrogenase 2 (IDH2), and components of the electron transport chain, to maintain oxidative phosphorylation efficiency [58,59,60]. SIRT6 acts on histone H3K9ac and H3K56ac to maintain heterochromatin and telomere integrity [61,62], while SIRT7 deacetylates histone H3K18ac and the transcription factor myocyte enhancer factor 2D (MEF2D), among others [41,63]. The dependence on NAD^+^ couples sirtuin activity directly to cellular metabolic state—a feature exploited by tumor cells through reprogramming of NAD^+^ biosynthesis [17,64].

### 2.3. Non-Canonical Activities: Desuccinylation, Demalonylation, and ADP-Ribosylation

An important development in sirtuin biology over the past decade, and especially since 2024, has been the recognition that sirtuins remove a much broader repertoire of acyl modifications than acetyl groups alone [20,46,65]. SIRT5 is the prototypical non-canonical sirtuin: it possesses minimal deacetylase activity but functions primarily as a desuccinylase, demalonylase, and deglutarylase, modifying mitochondrial enzymes including carbamoyl phosphate synthetase 1 and succinate dehydrogenase [20,65,66]. SIRT4 exhibits ADP-ribosyltransferase and lipoamidase activities, in addition to weak deacetylase activity, with ADP-ribosylation of glutamate dehydrogenase as its principal regulatory mechanism [21,67]. SIRT6 combines deacetylase activity with mono-ADP-ribosylation of substrates, including PARP1 [68,69].

One of the most notable recent findings for thyroid cancer is that SIRT7 functions as a desuccinylase with multiple oncogenic substrates, including KIF23 in anaplastic thyroid cancer and LATS1 in papillary thyroid cancer [24,25,27]. This dual activity (deacetylase and desuccinylase) has direct therapeutic implications: substrate-class-selective inhibitors that block oncogenic desuccinylation while sparing housekeeping deacetylase function represent an attractive concept for SIRT7-directed therapy (Section 7.3) [37]. The expansion of the sirtuin enzymatic repertoire beyond simple deacetylation is therefore not merely a biochemical curiosity but a foundational rationale for the drug development efforts reviewed in Section 7.

### 2.4. NAD^+^ Dependency and Metabolic Linkage

All seven sirtuins require NAD^+^ as a cofactor, coupling their activity to cellular NAD^+^ levels and thereby to metabolic state [49,64]. Cancer cells, including those of advanced thyroid cancer, frequently upregulate NAD^+^ biosynthesis through nicotinamide phosphoribosyltransferase (NAMPT)—the rate-limiting enzyme of the salvage pathway—to sustain elevated sirtuin activity [70]. Elevated NAMPT expression has been documented in ATC and contributes to the NAMPT-SIRT1-PD-L1 axis discussed in Section 5.2 [32,71]. This metabolic dependency creates pharmacological opportunities at multiple levels: NAMPT inhibitors deplete NAD^+^ pools and indirectly inhibit all sirtuins [72], while direct sirtuin inhibitors achieve isoform selectivity through interactions with the substrate-binding pocket [37]. The interface between NAD^+^ metabolism, sirtuin activity, and thyroid cancer biology is further developed in Section 4.5 and Section 7.

## 3. Dichotomous Roles of Sirtuins in Thyroid Cancer

**Figure 2:**
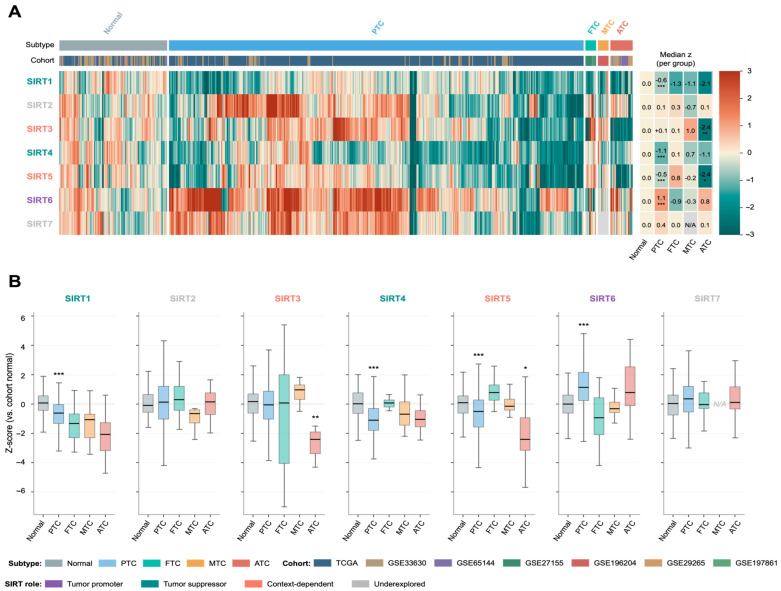
Differential expression of sirtuin family members across thyroid cancer subtypes. (**A**) Per-sample heatmap of SIRT1–SIRT7 z-scores (calculated against cohort-matched reference thyroid samples) in normal thyroid (*n* = 150), papillary thyroid cancer (PTC; n = 625, TCGA-THCA + GSE27155 + GSE29265 + GSE33630), follicular thyroid cancer (FTC; *n* = 17, GSE27155 + GSE197861), medullary thyroid cancer (MTC; *n* = 19, GSE27155 + GSE196264), and anaplastic thyroid cancer (ATC; *n* = 36, GSE27155 + GSE29265 + GSE33630 + GSE65144). Each column is one patient sample, ordered by hierarchical clustering within each subtype. The left annotation column indicates the established functional role of each SIRT in thyroid cancer based on the literature reviewed in Section 3 (purple = tumor promoter; teal = tumor suppressor; coral = context-dependent; gray = underexplored). The right-side summary panel shows the median z-score for each subtype with statistical significance vs. cohort-matched reference samples (Wilcoxon rank-sum test with Benjamini–Hochberg correction; * q < 0.05, ** q < 0.01, *** q < 0.001). Red shading indicates upregulation, teal indicates downregulation, and cream indicates no change relative to the reference thyroid. (**B**) Distribution of per-sample z-scores per SIRT gene across subtypes; box plots show the interquartile range and median, with individual sample points overlaid for groups with *n* ≤ 80. SIRT6 is significantly upregulated specifically in PTC, consistent with its proposed role in BRAF-driven proliferation. SIRT3, SIRT4, and SIRT5 are downregulated across most subtypes, most profoundly in ATC. SIRT1 paradoxically shows reduced mRNA expression across all subtypes despite protein-level upregulation reported in immunohistochemical studies (Section 3.1), highlighting the importance of post-translational regulation. SIRT2 and SIRT7 exhibit modest or non-significant changes (Wilcoxon rank-sum test with Benjamini–Hochberg correction; * q < 0.05, ** q < 0.01, *** q < 0.001). SIRT7 was not present on the Illumina HumanHT-12 WG-DASL platform (GSE196264); the MTC × SIRT7 cell is therefore shown as N/A. Detailed cohort selection, sample processing, normalization, statistical analysis methodology, software versions, and the complete gene-level expression matrices are provided in Supplementary Methods and Data.

The seven mammalian sirtuins (SIRT1–7) display strikingly heterogeneous expression patterns and functional outputs in thyroid cancer (TC), with individual isoforms acting as either tumor promoters or suppressors depending on the cellular context, the histological subtype, and the underlying genetic alterations [18,23,24,26,27,28,30]. As summarized in Figure 1 and quantified across thyroid cancer subtypes in Figure 2, this dichotomy defies a single unifying model and instead points toward a context-dependent regulatory network. Because this integrative transcriptomic analysis combines TCGA RNA-seq data with several independent GEO microarray cohorts of differing platforms and sample sizes, the inter-subtype differences should be interpreted as qualitative directional trends rather than as precise effect-size comparisons; this is particularly important for the rare subtypes (FTC, MTC) with small sample sizes, and a cohort-level directional consistency analysis is provided in the Supplementary Methods. Table 1 consolidates the current evidence linking each SIRT isoform to its enzymatic activity, expression status, principal substrates, and clinical relevance in thyroid cancer. In this section, we systematically examine the tumor-promoting and tumor-suppressive sirtuins, then introduce the emerging paradigm of non-canonical PTM regulation, which has expanded current understanding of sirtuin biology [25,27,46].

### 3.1. Tumor-Promoting Sirtuins

SIRT1. SIRT1 is the most extensively studied sirtuin in thyroid cancer and is reported to be upregulated at the protein level in papillary (PTC) and follicular (FTC) thyroid carcinomas by immunohistochemical and Western blot studies [23,30,73,75]. Of note, however, SIRT1 mRNA expression in our integrative transcriptomic analysis (Figure 2) is paradoxically reduced across all thyroid cancer subtypes (median z = −0.62 in PTC, q < 0.001; −1.33 in FTC; −1.07 in MTC; −2.08 in ATC), with the most pronounced decrease in ATC. This mRNA-protein discordance is a recognized feature of sirtuin biology and is consistent with the well-established observation that sirtuin functional output is governed predominantly by post-transcriptional and post-translational mechanisms—including NAD^+^ cofactor availability, DBC1 binding, AMPK-mediated phosphorylation, SUMOylation, and ubiquitin-mediated protein stabilization—rather than by transcript abundance [22,37]. This caveat is detailed in Section 3.5 and has direct implications for biomarker design: protein-level (rather than mRNA-level) profiling will be required for SIRT1-guided patient stratification in future clinical applications. Accordingly, throughout this review and in Table 1, SIRT1 status in thyroid cancer is described at three distinct levels—mRNA (downregulated), protein (upregulated), and function (tumor-promoting)—to avoid the ambiguity inherent in a single summary term. The same principle is applied to the other sirtuins wherever mRNA, protein, and functional evidence diverge. An influential study by Herranz and colleagues demonstrated that SIRT1 cooperates with PTEN deficiency to drive thyroid carcinogenesis: in *Pten*-null mouse models, Sirt1 haploinsufficiency markedly delayed tumor onset, while pharmacological SIRT1 activation by resveratrol or SRT1720 accelerated progression and increased the incidence of poorly differentiated lesions [23]. This finding was particularly notable because it reframed SIRT1—initially proposed as a tumor suppressor in some hematologic contexts [76,77]—as a context-dependent oncogene whose effects depend on the underlying genetic background.

Mechanistically, SIRT1 deacetylates and modulates the activity of multiple tumor suppressors, including p53 [51,52], FOXO1, FOXO3 [53,78], and Ku70 [79], thereby blunting apoptotic and DNA damage response programs that would otherwise restrain transformed cell growth. In thyroid follicular cells, SIRT1-mediated p53 deacetylation reduces transcription of pro-apoptotic targets such as PUMA and BAX, while FOXO3 deacetylation diminishes transcription of cell cycle inhibitors p21 and p27 [23,73]. SIRT1 induction also confers resistance to etoposide-induced genotoxic apoptosis in thyroid cancer cell lines, suggesting a direct contribution to chemoresistance and supporting the rationale for combining SIRT1 inhibitors with conventional chemotherapeutics in advanced disease [73]. Beyond these classical substrates, SIRT1 has been implicated in deacetylating the androgen receptor [80], NF-κB p65 [54], and HIF-1α [56] in other cancer contexts, increasing the possibility that SIRT1 in thyroid cancer also engages a broader substrate network than has been formally characterized.

Clinically, elevated SIRT1 expression in PTC correlates with adverse pathological features, including extrathyroidal extension, lymph node metastasis, and *BRAF* V600E status, although prospective validation of SIRT1 as an independent prognostic biomarker in adequately powered cohorts is still needed [30,73]. Whether SIRT1 expression predicts response to RAI therapy or to multikinase inhibitor treatment is currently unknown but represents an actionable research question given the emerging interest in SIRT1 inhibitors discussed in Section 7.

SIRT6. SIRT6 has a prominent role in *BRAF* V600E-driven thyroid cancer biology. Qu et al. reported that SIRT6 is significantly upregulated in PTC tissues and that its expression correlates with cancer aggressiveness; functionally, SIRT6 promotes proliferation and invasion by activating the BRAF/ERK/Mcl-1 axis [26]. Subsequent work by Yang and colleagues established SIRT6 as an important contributor to EMT in PTC by stabilizing HIF-1α under hypoxic conditions, thereby increasing expression of mesenchymal markers and reducing E-cadherin [28]. A complementary study further demonstrated that SIRT6 promotes the Warburg effect in PTC via reactive oxygen species (ROS)-mediated signaling [81], linking SIRT6 to the metabolic reprogramming axis described in Section 4.5. The convergence of these axes—proliferation/survival via BRAF/ERK, invasion/metastasis via HIF-1α, and Warburg-like metabolism—positions SIRT6 as a particularly rational therapeutic target in aggressive PTC and anaplastic thyroid cancer (ATC). Recent ferroptosis literature has further implicated SIRT6 as an important regulator of redox homeostasis in ATC [33], a topic developed in Section 6.

SIRT7. SIRT7 has emerged as a context-dependent regulator with predominantly tumor-promoting effects in thyroid cancer. Li and colleagues demonstrated that SIRT7 promotes thyroid tumorigenesis by enhancing AKT and p70S6K1 phosphorylation through a DBC1/SIRT1 axis [24]. More recently, two studies have repositioned SIRT7 within a non-canonical deacylation framework: Wu et al. showed that SIRT7 regulates KIF23 desuccinylation and supports ATC cell viability and migration [27], while Li and Pu reported that SIRT7 interacts with LATS1 and reduces LATS1 stability through desuccinylation in PTC models [25]. The SIRT7–KIF23 finding currently rests on a single primary study and awaits independent replication; it should therefore be interpreted as a promising but not yet independently confirmed ATC oncogenic mechanism. Because LATS1 is a core upstream kinase in the Hippo tumor-suppressor pathway, SIRT7-mediated LATS1 destabilization may attenuate Hippo pathway output, although downstream YAP/TAZ activation has not yet been directly demonstrated [25,82,83]. These findings, discussed in detail in Section 3.3, fundamentally expand SIRT7’s mechanistic repertoire beyond classical deacetylation [32,84].

### 3.2. Tumor-Suppressive Sirtuins

In contrast to the nuclear sirtuins discussed above, the mitochondrial sirtuins SIRT3 and SIRT4 are predominantly downregulated in differentiated thyroid cancer (DTC) and exert tumor-suppressive functions through control of mitochondrial metabolism and redox balance [29,30,46,74].

SIRT3 is the principal mitochondrial deacetylase and regulates oxidative phosphorylation (OXPHOS) by deacetylating substrates such as SOD2 [59,85] and IDH2 [60]. Bioinformatic analysis of TCGA data has shown significant SIRT3 downregulation in DTC, with loss of SIRT3 expression associated with increased reactive oxygen species (ROS) and metabolic reprogramming toward aerobic glycolysis [30,86]. The Warburg-like shift consequent to SIRT3 loss is consistent with the metabolic phenotype of dedifferentiated and ATC tumors discussed in Section 6 [87,88].

**Figure 3:**
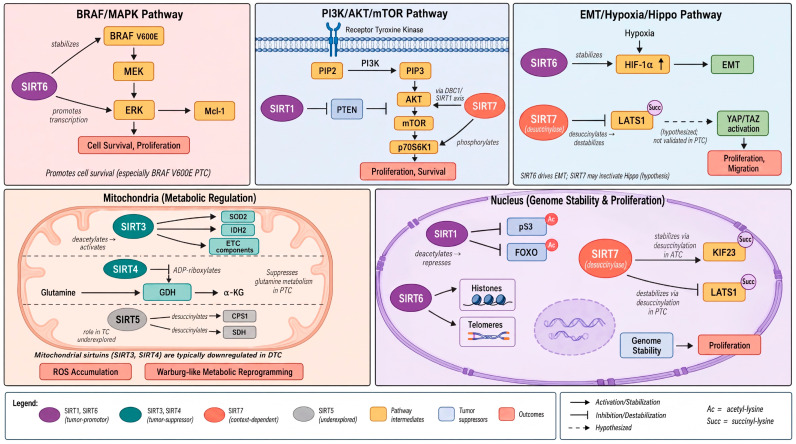
SIRT-mediated signaling and metabolic pathways in thyroid cancer. Sirtuins regulate five interconnected axes that drive thyroid cancer progression. In the BRAF/MAPK pathway (**top left**), SIRT6 amplifies BRAF/ERK signaling and promotes Mcl-1 expression, sustaining cell survival, particularly in *BRAF* V600E-mutant papillary thyroid cancer (SIRT6 acts both upstream and downstream of BRAF to sustain MAPK output; direct stabilization of the *BRAF* V600E protein itself has not been demonstrated). In the PI3K/AKT/mTOR pathway (**top middle**), SIRT1 deacetylates and represses PTEN (which normally dephosphorylates PIP3 to PIP2), thereby enhancing AKT activation, while SIRT7 promotes AKT/p70S6K1 phosphorylation via the DBC1/SIRT1 axis. In the EMT/Hypoxia/Hippo pathway (**top right**), SIRT6 stabilizes HIF-1α (↑ denotes increased expression) to drive EMT, while SIRT7 desuccinylates and destabilizes LATS1; the downstream YAP/TAZ activation in PTC remains hypothesis-generating and is depicted with a dashed arrow. In mitochondria (**bottom left**), the tumor-suppressive sirtuins SIRT3 (deacetylating SOD2 and IDH2) and SIRT4 (ADP-ribosylating and inhibiting GDH, thereby limiting glutamine flux to α-ketoglutarate) are typically downregulated in differentiated thyroid cancer, leading to ROS accumulation and a Warburg-like metabolic reprogramming. SIRT5, which functions as a desuccinylase regulating CPS1 and SDH, is shown in grey to indicate its underexplored role in thyroid cancer. In the nucleus (**bottom right**), SIRT1 deacetylates and represses p53 and FOXO, and SIRT6 modulates histone acetylation and telomere integrity, jointly regulating genome stability; SIRT7 stabilizes KIF23 in ATC (via desuccinylation) and destabilizes LATS1 in PTC (via desuccinylation), representing an emerging non-canonical PTM paradigm (2024–2025). Together, these axes converge on increased proliferation, migration, dedifferentiation, and radioiodine (RAI) refractoriness.

SIRT4 has been characterized as a well-supported tumor suppressor in PTC through its capacity to inhibit glutamine metabolism. SIRT4 ADP-ribosylates and represses glutamate dehydrogenase (GDH), limiting the entry of glutamine-derived carbon into the tricarboxylic acid cycle [21,74]. Lee et al. demonstrated that SIRT4 expression is downregulated in 205 PTC tissue samples and that low SIRT4 expression correlates with reduced overall survival in TCGA and increased extracapsular extension (*p* < 0.001) [89]. SIRT4 reconstitution in PTC cell lines (B-CPAP, TPC-1, SNU-790) suppresses proliferation and invasion both in vitro and in B-CPAP xenograft models, with concurrent induction of mitochondrial ROS-mediated apoptosis and suppression of epithelial–mesenchymal transition (E-cadherin upregulation; N-cadherin, MMP9, MMP3, and SNAIL downregulation) [89]. Recent metabolic reprogramming reviews highlight glutamine addiction as a targetable vulnerability in BRAF-mutant thyroid cancer [74,90]. The pathway-level integration of SIRT3 and SIRT4 with mitochondrial metabolism is further developed in Section 4.5 and visualized in Figure 3 (bottom left).

SIRT2 and SIRT5 remain underexplored in thyroid cancer despite established roles in other malignancies. SIRT2 is primarily cytoplasmic and regulates mitotic processes through tubulin deacetylation [42,48], while SIRT5 is the principal mitochondrial desuccinylase, demalonylase, and deglutarylase [20,65,66]. Bioinformatic surveys suggest both are downregulated in DTC [30], but mechanistic studies in thyroid models are largely lacking—a gap that we revisit as a research priority in Section 10. Of note, our integrative transcriptomic analysis (Figure 2) reveals that SIRT5 mRNA is significantly downregulated in ATC (median z = −2.42)—a magnitude comparable to that observed for the better-characterized tumor-suppressive mitochondrial sirtuins SIRT3 (z = −2.42) and SIRT4 (z = −1.05) in the same subtype. This previously underappreciated finding suggests that ATC may exhibit a coordinated collapse of multiple mitochondrial sirtuins (SIRT3, SIRT4, and SIRT5), extending the mitochondrial-sirtuin-loss paradigm beyond the canonical SIRT3-SOD2/IDH2 and SIRT4-GDH axes. Given that SIRT5 controls succinylation, malonylation, and glutarylation of TCA cycle and electron transport chain enzymes—including carbamoyl phosphate synthetase 1 (CPS1), succinate dehydrogenase (SDH), and acyl-CoA dehydrogenases [20,65,66]—its loss in ATC may contribute substantially to the metabolic dysfunction that defines this most aggressive thyroid cancer subtype. Because this represents a transcript-level signal without corresponding protein-level or functional validation in thyroid cancer, it should be regarded as a hypothesis requiring dedicated experimental confirmation (see Section 10).

### 3.3. Non-Canonical Post-Translational Modification Regulation: An Emerging Paradigm

For most of the past two decades, research on sirtuins in cancer has focused almost exclusively on their NAD^+^-dependent deacetylase activity [49,50]. However, since 2024, the field has been reshaped by a growing recognition that several sirtuins—most notably SIRT5 and SIRT7—can remove a diverse repertoire of acyl modifications, including succinyl, malonyl, and glutaryl groups [20,46,65]. In thyroid cancer, two pivotal studies have established desuccinylation as a bona fide regulatory mechanism with direct oncogenic relevance.

Wu et al. identified KIF23 as a functionally relevant substrate of SIRT7-mediated desuccinylation in an ATC-focused thyroid cancer cell study [27]. KIF23 was upregulated in the cell models examined, and KIF23 knockdown reduced cell viability and migration. Mechanistically, SIRT7 interacted with KIF23 and reduced KIF23 succinylation, with K537 identified as a candidate desuccinylation site in HEK-293T-based mutational assays. SIRT7 overexpression increased KIF23 protein stability, whereas SIRT7 silencing attenuated KIF23-driven viability and migration phenotypes. However, K537 desuccinylation was not confirmed by mass spectrometry, and in vivo validation has not yet been performed. Thus, the SIRT7–KIF23 axis should currently be regarded as a promising, yet still preclinical, therapeutic axis in ATC [27].

Building on this discovery, Li and Pu reported that SIRT7 interacts with LATS1, a core kinase of the Hippo tumor-suppressor pathway, and reduces LATS1 protein stability through desuccinylation in PTC models [25]. SIRT7 silencing increased LATS1 succinylation and stability, suppressed PTC cell proliferation, promoted apoptosis, and reduced tumor growth in a TPC-1 xenograft model [25]. Because LATS1 is an upstream Hippo pathway kinase that restrains YAP/TAZ-dependent oncogenic transcription, these findings suggest that SIRT7-mediated LATS1 destabilization may weaken Hippo tumor-suppressor signaling [82,83]. However, direct evidence for reduced LATS1 kinase activity, altered YAP/TAZ phosphorylation, YAP/TAZ nuclear accumulation, or induction of canonical YAP/TAZ target genes in this SIRT7–LATS1 setting remains lacking. Therefore, the SIRT7–LATS1 axis should be presented as a promising PTC oncogenic mechanism with a plausible, but not yet fully demonstrated, connection to downstream YAP/TAZ signaling [25].

These studies converge on a unified concept: SIRT7 functions as an oncogenic desuccinylase across the spectrum of thyroid cancer, from PTC to ATC, with substrate specificity that varies by cellular context. They also raise the broader question of whether other sirtuins—particularly SIRT5, which has well-established desuccinylase activity in metabolic contexts [20,66]—exert similar non-canonical regulatory effects in thyroid cancer that have not yet been characterized. We anticipate that the next several years will see a substantial expansion of the non-canonical SIRT-substrate landscape in thyroid cancer [22,91].

### 3.4. Subtype-Specific Expression Patterns

The dichotomous role classification developed above must be interpreted in the context of histological heterogeneity. As shown in Figure 2, SIRT mRNA expression patterns vary substantially across thyroid cancer subtypes and do not conform to a single monotonic progression. SIRT6 mRNA is most strongly upregulated specifically in PTC (median z = +1.14, q < 0.001), consistent with its established role as a BRAF/ERK pathway amplifier in *BRAF* V600E-driven PTC [26,28]; this PTC-specific peak, rather than a PTC-to-ATC progressive increase, suggests that SIRT6 functional engagement is linked to the active BRAF/MAPK output that defines PTC, while in ATC—where MAPK signaling is intact but additional drivers such as TP53 loss dominate—SIRT6 transcript levels return toward baseline. SIRT3 and SIRT4 show pronounced mRNA downregulation in ATC (median z scores of −2.42 and −1.05, respectively), consistent with the loss of normal mitochondrial differentiation in aggressive disease and the metabolic shift toward aerobic glycolysis that characterizes ATC [74,92]. SIRT5 shows a comparable downregulation in ATC (Section 3.2), consistent with the coordinated loss of mitochondrial sirtuins in this subtype. SIRT1 mRNA, in contrast, is downregulated across all subtypes—most profoundly in ATC (median z = −2.08)—despite consistent protein-level upregulation reported in immunohistochemical studies (Section 3.1). SIRT7 mRNA shows minimal subtype-specific variation across the analyzed cohorts, consistent with our overall view of SIRT7 as a substrate-level (rather than transcription-level) regulator whose oncogenic activity in thyroid cancer is primarily mediated by desuccinylation of specific substrates such as KIF23 and LATS1 (Section 3.3) [25,27]. The transcript-protein discordance for SIRT1 and the substrate-level mechanism for SIRT7 collectively underscore that mRNA expression cannot be used as a stand-alone proxy for sirtuin functional activity—a methodological caveat developed in Section 3.5. The overall pattern—concurrent loss of multiple mitochondrial sirtuins (SIRT3, SIRT4, SIRT5) in ATC and PTC-specific elevation of SIRT6—supports an integrated SIRT-stratification framework for therapeutic decisions, which is further developed in Section 8.

Medullary thyroid cancer (MTC), which arises from parafollicular C-cells rather than thyroid follicular epithelium, displays a distinct expression profile that remains poorly characterized due to its rarity (approximately 3–5% of thyroid cancers) [92]. Limited bioinformatic surveys suggest that SIRT3 and SIRT6 may show modest upregulation in MTC, but whether these patterns reflect true biological differences or are due to sample size limitations is unclear [30]. Given that MTC is driven by RET mutations (germline in MEN2 syndromes, somatic in sporadic cases) rather than BRAF or RAS, the relevant SIRT-regulated pathways may differ substantially from those in DTC [92,93].

*A note on medullary thyroid cancer.* Because medullary thyroid cancer (MTC) arises from parafollicular C-cells rather than follicular epithelial cells and is driven by RET-activating mutations rather than BRAF or RAS, MTC biology cannot be simply mapped onto the SIRT framework developed here for follicular-derived DTC and ATC. Direct evidence linking individual sirtuins to MTC pathogenesis remains very limited, and the specific RET-driven downstream networks (e.g., RAS/MAPK, PI3K/AKT, calcitonin secretion regulation) that may intersect with sirtuin biology have not been systematically characterized. References to MTC in this review should therefore be interpreted as illustrative of the broader thyroid cancer landscape rather than as a validated SIRT-MTC mechanistic narrative, and dedicated MTC-focused studies are needed before SIRT-based strategies can be considered in this subtype.

The convergence of upregulated tumor-promoter sirtuins and downregulated tumor-suppressor sirtuins in ATC underscores the potential value of SIRT-targeted therapy in this clinical context, where treatment options remain severely limited despite recent advances with the BRAF/MEK combination and the emergence of ICIs [9,10,15]. Patient stratification based on integrated SIRT expression profiles—for instance, identifying tumors with concurrent SIRT6/SIRT7 upregulation and SIRT3/SIRT4 loss—could enable targeted use of SIRT inhibitors in combination regimens, an approach we develop further in Section 8.

*Evidence levels for the principal SIRT–TC mechanistic axes.* To clarify the maturity of evidence supporting each axis discussed below, Table 2 summarizes the evidence type, thyroid cancer specificity, in vivo support, clinical correlation, and current therapeutic readiness for the major SIRT–TC mechanisms. The SIRT6–BRAF/ERK/Mcl-1 and SIRT7–LATS1 axes have the strongest thyroid cancer-specific support with cell line and xenograft validation, whereas the SIRT7–KIF23, SIRT6–NCOA4 ferroptosis, and NAMPT–SIRT1–PD-L1 axes remain at earlier preclinical or hypothesis-generating stages. This evidence stratification is important for interpreting the combination strategies discussed in Section 8 and the clinical translation outlook in Section 10.

### 3.5. Transcript-Level Patterns: Confirmation, Contrast, and a Methodological Caveat

The transcriptomic patterns presented in Figure 2 should be interpreted within an evidence-stratification framework that explicitly recognizes three distinct layers of evidence in sirtuin biology: (i) functional/genetic evidence, derived from gain-of-function, loss-of-function, and knockout studies in cellular and animal models; (ii) protein-level expression evidence, derived from immunohistochemistry, Western blot, and proteomic analyses of patient tumor samples; and (iii) transcript-level expression evidence, derived from RNA-sequencing or microarray analyses of patient cohorts. These three layers can yield concordant or discordant signals depending on the specific sirtuin and the cellular context, and conflating them can lead to apparent contradictions in the literature [22,37].

Figure 2 illustrates this stratification explicitly. The PTC-specific SIRT6 upregulation observed at the mRNA level (median z = +1.14, q < 0.001) is concordant with the protein-level findings reported by Qu et al. and Yang et al. and with the functional evidence linking SIRT6 to the BRAF/ERK/Mcl-1 axis and HIF-1α-driven EMT [26,28,81]—a case where all three layers converge on the same conclusion. Similarly, the pronounced ATC downregulation of SIRT3 and SIRT4 (median z scores of −2.42 and −1.05, respectively) parallels protein-level findings and supports their tumor-suppressive roles in mitochondrial metabolism [30,74,86]. In these cases, the transcript-level data in Figure 2 provide confirmatory support for the functional and protein-level evidence reviewed in Section 3.1 and Section 3.2.

However, three observations in Figure 2 explicitly contrast with, or fail to recapitulate, the protein-level and functional evidence. First, SIRT1 mRNA is reduced rather than elevated across all thyroid cancer subtypes, in apparent contrast to the IHC and Western blot evidence reporting SIRT1 protein elevation in PTC and FTC [23,30,73,75]. This discordance is mechanistically explainable: SIRT1 protein abundance and activity are determined predominantly by post-transcriptional and post-translational mechanisms—including NAD^+^ cofactor availability, DBC1 binding (which sequesters SIRT1 in an inactive complex), AMPK-mediated phosphorylation, SUMOylation, and ubiquitin-mediated stability control—rather than by transcriptional regulation [22,37]. Second, SIRT7 mRNA shows minimal subtype-specific variation, despite strong functional evidence placing SIRT7 as an oncogenic regulator in both PTC and ATC, consistent with its established role as a substrate-level (rather than expression-level) regulator that exerts its effects through targeted desuccinylation of specific substrates such as KIF23 and LATS1 [25,27]. Third, the SIRT5 ATC downregulation (Section 3.2) is a transcript-level signal lacking corresponding protein-level or functional validation in thyroid cancer and therefore exemplifies a transcript-only hypothesis requiring dedicated experimental confirmation (Section 10).

These observations collectively support a methodological caveat that has direct implications for biomarker design and clinical translation: transcript expression alone is insufficient to define sirtuin functional status in thyroid cancer. Three practical consequences follow. First, biomarker development should prioritize protein-level (IHC, mass spectrometry-based proteomics) over mRNA-level profiling, particularly for SIRT1 and SIRT7, where the discordance is most pronounced. Second, integrative biomarker panels combining SIRT protein expression with mutational status (*BRAF* V600E, PTEN, TP53) and substrate-level PTM markers (e.g., LATS1 succinylation, KIF23 succinylation, FOXP3 acetylation status) are likely to outperform any single-marker approach. Third, the bioinformatic analysis presented here should be regarded as a directional summary of the transcriptomic evidence base rather than as a substitute for protein-level validation studies, and the rare-subtype findings (FTC, MTC, ATC) should be interpreted as hypothesis-generating, given the modest sample sizes and platform heterogeneity (see Supplementary Methods and Data). This three-layer evidence framework is referenced throughout Section 4 and Section 5 when describing the mechanistic axes and is foundational to the clinical translation strategies developed in Section 8.

## 4. Sirtuins in Key Signaling and Metabolic Pathways of Thyroid Cancer

Building on the dichotomous role framework established in Section 3, this section examines the specific signaling and metabolic axes through which sirtuins exert their effects in thyroid cancer. Figure 3 provides an integrated overview of these pathways, organized into five interconnected panels covering BRAF/MAPK signaling, PI3K/AKT/mTOR signaling, EMT and the Hippo pathway, mitochondrial metabolism, and genome stability.

### 4.1. BRAF/MAPK Pathway

The *BRAF* V600E mutation, present in approximately 60% of PTC cases [94,95], is the most prevalent oncogenic driver in thyroid cancer and constitutively activates the RAS-RAF-MEK-ERK cascade, leading to sustained proliferation and survival signaling [26,96]. SIRT6 has been identified as a major amplifier of *BRAF* V600E signaling in this context. Qu and colleagues demonstrated that SIRT6 expression is elevated in PTC tissues and correlates with *BRAF* V600E status; in BRAF-mutant PTC cell lines, SIRT6 knockdown reduces ERK phosphorylation, decreases Mcl-1 expression, and sensitizes cells to apoptosis, while SIRT6 overexpression has the opposite effect [26]. Mechanistically, SIRT6 appears to act both upstream and downstream of BRAF: it stabilizes activated forms of RAF kinase and promotes transcription of Mcl-1, providing a dual mechanism by which SIRT6 sustains MAPK output in BRAF-mutant cells [26,28].

The therapeutic implication is significant. Single-agent BRAF inhibitors (vemurafenib, dabrafenib) achieve modest response rates in advanced BRAF-mutant PTC and are limited by the rapid emergence of MAPK reactivation through compensatory mechanisms, including HER3 upregulation [97], KRAS activating mutations [98,99], and BRAF splice variants [100,101,102]. Combination with the MEK inhibitor trametinib improves outcomes but does not overcome eventual resistance [9,10]. SIRT6 inhibition could potentiate BRAF inhibitors by attenuating downstream ERK output and reducing Mcl-1-dependent survival and could also pre-empt MAPK reactivation in BRAF-resistant settings. Although no SIRT6 inhibitor has yet entered clinical trials in thyroid cancer, the small molecule OSS_128167 has shown promising preclinical activity in other tumor models [103,104] and represents a candidate for thyroid cancer-focused development. This combination strategy is explored further in Section 8.

### 4.2. PI3K/AKT/mTOR Pathway

The PI3K/AKT/mTOR axis is hyperactivated in approximately 30–40% of thyroid cancers, with PTEN loss representing the most common upstream alteration, particularly in FTC and ATC [94,105,106]. Two sirtuins converge on this pathway through distinct mechanisms (Figure 3, top middle). SIRT1 deacetylates PTEN at multiple lysine residues, promoting its nuclear export and proteasomal degradation; in *Pten*-haploinsufficient backgrounds, SIRT1 elevation amplifies the consequences of partial PTEN loss and accelerates thyroid carcinogenesis [23,107]. This SIRT1-PTEN axis is particularly relevant for PI3K-driven FTC and for ATC, where combined PTEN and TP53 loss is a frequent transformation event [94,108]. Downstream of AKT, SIRT7 enhances p70S6K1 phosphorylation through a DBC1/SIRT1 axis, providing a second SIRT-dependent route to mTOR activation and to translation of pro-proliferative and pro-survival mRNAs [24].

Beyond these primary axes, secondary contributions from other sirtuins likely modulate PI3K/AKT signaling in thyroid cancer. SIRT3 loss in DTC has been associated with increased AKT phosphorylation in some contexts, possibly through ROS-mediated activation of upstream tyrosine kinases [86,87], while SIRT6 may indirectly influence PI3K signaling through HIF-1α-mediated transcription of growth factor receptors [28,109]. These secondary axes have not yet been formally characterized in thyroid cancer models and represent avenues for future mechanistic work.

The dual involvement of SIRT1 and SIRT7 in the PI3K/AKT/mTOR pathway suggests that combined SIRT inhibition may provide synergistic benefit, particularly in PTEN-deficient or PI3K-driven thyroid cancers. However, isoform selectivity and on-target toxicity remain key development challenges, especially given SIRT1’s broader physiological roles in metabolic homeostasis and longevity [22,37] (Section 7). Clinical translation will require careful patient stratification, ideally guided by predictive biomarkers reflecting PI3K pathway activation status and SIRT expression.

### 4.3. EMT and Metastasis

EMT is a central driver of metastasis in thyroid cancer, particularly in aggressive PTC variants (tall cell, columnar cell), poorly differentiated thyroid cancer, and ATC [28,110]. SIRT6 promotes EMT through HIF-1α stabilization: under hypoxic conditions or in response to oncogenic BRAF signaling, SIRT6 deacetylates and stabilizes HIF-1α, increasing transcription of master EMT transcription factors including Snail, Slug, and ZEB1, with subsequent loss of epithelial markers (E-cadherin, claudins) and gain of mesenchymal markers (N-cadherin, vimentin) [28,109]. SIRT6 knockdown reverses this phenotype and reduces invasion in PTC cell lines and xenograft models [28], supporting a role for SIRT6 as an EMT regulator in thyroid cancer.

Beyond SIRT6, other sirtuins likely contribute to EMT through pathway-specific mechanisms. SIRT1 has been shown to deacetylate Smad4 and Snail in other epithelial cancers, stabilizing these EMT-promoting factors and enhancing TGF-β signaling [111,112]. Similar mechanisms may operate in thyroid cancer, although direct evidence is limited. Conversely, SIRT3 loss promotes EMT indirectly by activating NF-κB and stabilizing Snail via ROS [87,113], suggesting that mitochondrial sirtuin downregulation in DTC contributes to mesenchymal transition. The integration of these multiple SIRT inputs into the EMT program in thyroid cancer remains poorly defined and warrants further mechanistic investigation.

The SIRT6/HIF-1α/EMT axis is particularly relevant for clinically aggressive disease characterized by lymphovascular invasion and distant metastasis and represents a candidate target for combination with multikinase inhibitors with anti-angiogenic activity (lenvatinib, sorafenib) [12,13]. A combination of SIRT6 inhibitors with HIF-1α-targeted agents or anti-angiogenic therapies could provide a complementary attack on the EMT and metastatic phenotype [28], an approach warranting exploration in preclinical models of advanced thyroid cancer.

### 4.4. Hippo/LATS1 Pathway: An Emerging Axis

The Hippo pathway has recently been implicated in thyroid cancer biology [82,114], and the identification of SIRT7-mediated LATS1 desuccinylation provides an emerging mechanistic link between sirtuin activity and Hippo pathway regulation in PTC [25]. In PTC models, SIRT7 interacts with LATS1 and reduces LATS1 stability through desuccinylation, whereas SIRT7 silencing increases LATS1 succinylation and stability, suppresses cell proliferation, promotes apoptosis, and reduces tumor growth in a TPC-1 xenograft model [25]. Since LATS1 is an upstream kinase that can restrain YAP/TAZ-dependent oncogenic transcription, SIRT7-mediated reduction in LATS1 stability may attenuate Hippo tumor-suppressor signaling [82,83]. However, the available PTC study did not directly demonstrate decreased LATS1 kinase activity, altered YAP/TAZ phosphorylation, nuclear accumulation of unphosphorylated YAP/TAZ, or induction of canonical YAP/TAZ target genes. Thus, LATS1 succinylation status represents a potential biomarker for SIRT7-directed therapy, whereas the downstream YAP/TAZ mechanism should currently be considered hypothesis-generating and requires direct validation in thyroid cancer models [25].

### 4.5. Metabolic Reprogramming

Thyroid cancer cells, particularly those of dedifferentiated and anaplastic histology, undergo extensive metabolic reprogramming characterized by increased aerobic glycolysis, glutamine addiction, and altered lipid metabolism [29,74,115]. Mitochondrial sirtuins are closely linked to this reprogramming.

SIRT3 maintains OXPHOS efficiency through deacetylation of SOD2 [59,85], IDH2 [60], and components of the electron transport chain [58,116]. SIRT3 loss, observed in DTC and especially in ATC, leads to ROS accumulation, mitochondrial dysfunction, and a shift toward aerobic glycolysis—the metabolic hallmark of aggressive thyroid cancer [30,86,88].

SIRT4 specifically inhibits glutamine metabolism through ADP-ribosylation of GDH [21,67]. Glutamine addiction is a particularly prominent feature of *BRAF* V600E-mutant thyroid cancer, where activated MAPK signaling drives glutaminolysis to support biosynthesis and redox homeostasis [74,90]. SIRT4 reconstitution restrains this glutamine flux and suppresses tumor growth, suggesting that SIRT4 activators or glutaminase inhibitors (e.g., CB-839/telaglenastat) could synergize with BRAF inhibitors in clinical settings [74,117].

SIRT5 is the principal mitochondrial desuccinylase, demalonylase, and deglutarylase, and modulates the activity of metabolic enzymes, including carbamoyl phosphate synthetase 1 (CPS1) and succinate dehydrogenase (SDH) [20,65,66]. Despite its established role in cancer metabolism in other contexts [118,119], the contribution of SIRT5 to thyroid cancer metabolic reprogramming remains essentially uncharacterized—a notable gap given the prominence of metabolic alterations in advanced thyroid cancer.

The metabolic reprogramming axis intersects directly with ferroptosis sensitivity, a topic developed in Section 6, where loss of mitochondrial sirtuins contributes to the dedifferentiation phenotype and RAI refractoriness that define the most clinically challenging thyroid cancers [33,34].

### 4.6. Genome Stability and DNA Repair

Sirtuins also regulate genome stability by deacetylating histone and non-histone substrates involved in the DNA damage response [19,120]. SIRT1 deacetylates p53 and FOXO transcription factors to modulate DNA damage signaling [52,53], while SIRT6 maintains heterochromatin and telomere integrity through deacetylation of H3K9 and H3K56 [61,62,121]. In thyroid cancer, where dedifferentiation and chromosomal instability accelerate the transition from DTC to ATC, dysregulation of these genome-protective functions likely contributes to disease progression [108,122].

The most striking recent development in this domain is the recognition that SIRT7 regulates non-histone substrates—KIF23 in ATC and LATS1 in PTC—via desuccinylation [25,27]. As discussed in Section 3.3, these findings reposition SIRT7 as a non-canonical PTM regulator with distinct substrates in different thyroid cancer subtypes and suggest that the full spectrum of SIRT-regulated proteins involved in genome stability and proliferation may be considerably larger than currently appreciated [32,46,84]. This expanding mechanistic repertoire provides a rationale for the drug development efforts reviewed in Section 7 and the combination strategies proposed in Section 8.

## 5. Sirtuins in the Thyroid Tumor Immune Microenvironment

The development of ICIs has fundamentally changed the treatment landscape for many solid tumors [123,124], but their impact on thyroid cancer remains modest, with overall response rates below 30% and substantial heterogeneity across histological subtypes [15,16,32]. Among thyroid cancers, anaplastic thyroid cancer (ATC) shows greater immunotherapy sensitivity than advanced papillary thyroid cancer (PTC), reflecting differences in tumor mutational burden, PD-L1 expression, and immune cell infiltration [15,125,126]. As detailed below, sirtuins emerge as critical regulators of these immune microenvironment features and represent a promising avenue for rational combination therapy with ICIs, particularly in the substantial fraction of patients who do not respond to current immunotherapy regimens [31,32]. It should be emphasized at the outset that direct evidence for SIRT-mediated immune regulation specifically in thyroid cancer is currently very limited. The three principal immune axes discussed in this section—the NAMPT–SIRT1–PD-L1 axis, SIRT6-related regulatory T-cell biology, and SIRT2 as a T-cell metabolic checkpoint—are derived predominantly from studies in non-thyroid cancers (melanoma, non-small-cell lung cancer, colorectal cancer, and hematologic malignancies; evidence from other cancer types). Their relevance to the thyroid cancer immune microenvironment is therefore presented as a set of biologically grounded but testable hypotheses requiring dedicated validation in thyroid cancer models, rather than as established thyroid cancer mechanisms.

### 5.1. The Immune-Cold vs. Immune-Hot Framework

Recent integrative reviews have proposed that isoform-specific sirtuin axes define the immunological state of tumors [31,32]. In this framework, certain sirtuins promote an immunosuppressive (“immune-cold”) tumor microenvironment characterized by regulatory T cell (Treg) accumulation, M2-polarized tumor-associated macrophages (TAMs), myeloid-derived suppressor cell (MDSC) recruitment, and elevated immune checkpoint expression, while others support an immunoreactive (“immune-hot”) state with active CD8^+^ T cell cytotoxicity, M1-polarized macrophages, and natural killer cell engagement [127,128]. The dichotomous functions of sirtuins in tumor immunity parallel their context-dependent roles in cancer cell-intrinsic biology (Section 3), but the substrate networks and molecular mechanisms are largely independent [31,32].

**Figure 4:**
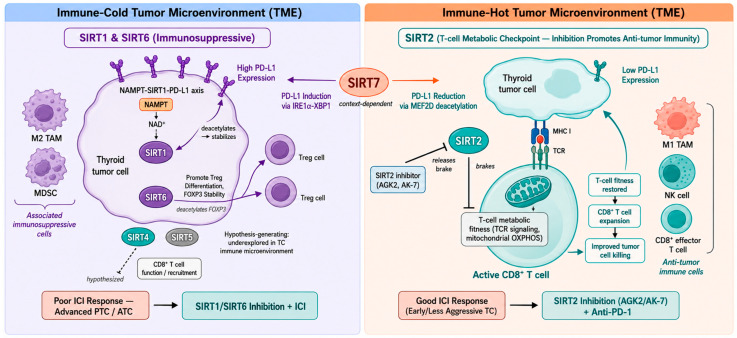
SIRT axes in the thyroid tumor immune microenvironment. Isoform-specific sirtuin axes determine the immunological state of thyroid tumors. On the immune-cold side (**left**, purple), SIRT1 stabilizes PD-L1 through the NAMPT-SIRT1-PD-L1 axis (NAMPT provides NAD^+^ as cofactor for SIRT1, which deacetylates and stabilizes PD-L1), SIRT6 supports regulatory T cell (Treg) differentiation and FOXP3 stability via deacetylation, with associated immunosuppressive cells (M2 TAM, MDSC) accumulating in the tumor microenvironment. SIRT4 (teal) and SIRT5 (grey) are depicted with dashed arrows to indicate their hypothesis-generating, underexplored roles in the TC immune microenvironment. Together, these axes create an immunosuppressive tumor microenvironment with elevated levels of Tregs, M2 TAMs, MDSCs, and PD-L1. On the immune-hot side (**right**, teal), SIRT2 functions as a metabolic checkpoint (brake) in tumor-reactive CD8^+^ T cells; SIRT2 inhibition (using tool inhibitors AGK2 or AK-7) releases this brake, restoring T-cell receptor signaling fitness, mitochondrial oxidative phosphorylation, and CD8^+^ T cell expansion, with concurrent M1 macrophage polarization [129]. SIRT7 (coral, context-dependent) plays dual roles: IRE1α-XBP1-mediated PD-L1 induction (immunosuppressive contribution) or MEF2D deacetylation-mediated PD-L1 reduction (immunoactivating contribution), depending on cellular context. Therapeutic strategies fall into two categories: SIRT1/SIRT6 inhibition combined with immune checkpoint inhibitors (ICIs) for cold tumors (e.g., advanced PTC, ATC), and SIRT2 inhibition (using AGK2, AK-7) combined with anti-PD-1 therapy to enhance tumor-reactive CD8^+^ T-cell metabolic fitness in hot tumors. Among thyroid cancer subtypes, ATC paradoxically combines features of both immune-cold and immune-hot microenvironments (high dedifferentiation, high PD-L1 expression, and TLS formation), making it the most ICI-responsive subtype, whereas advanced PTC tends toward immune coldness. NAMPT, nicotinamide phosphoribosyltransferase; TAM, tumor-associated macrophage; MDSC, myeloid-derived suppressor cell; TLS, tertiary lymphoid structure; OXPHOS, oxidative phosphorylation. Arrows: → activation; ⊣ inhibition; dashed arrow = hypothesized or not-yet-validated relationship.

Within this framework, current evidence supports the following classification: SIRT1 and SIRT6 appear to function predominantly as immunosuppressive regulators within the TME (NAMPT–SIRT1–PD-L1 and SIRT6–Treg axes demonstrated in melanoma, NSCLC, and other non-thyroid models; not yet in thyroid cancer); SIRT2 functions as a T-cell metabolic checkpoint whose inhibition can enhance cytotoxic T-cell metabolic fitness and effector function (shown in melanoma and colorectal models [129]; thyroid cancer data lacking); SIRT4 and SIRT5 may impair CD8^+^ T cell function and recruitment, contributing to immune escape; and SIRT7 plays a context-dependent role with both immunosuppressive and immunoactivating effects depending on the substrate engaged [31,32,129]. In thyroid cancer, this immune-axis dichotomy intersects with the well-documented histological gradient of immune infiltration: ATC tumors display higher PD-L1 expression and tertiary lymphoid structure (TLS) formation than advanced PTC, partially explaining the differential ICI response rates observed clinically [15,125,126]. Figure 4 illustrates this immune-axis dichotomy in the context of thyroid cancer.

### 5.2. Immune-Suppressive Sirtuin Axes (SIRT1, SIRT6)

SIRT1 has been implicated in immunosuppression through the NAMPT–SIRT1–PD-L1 axis, in which nicotinamide phosphoribosyltransferase (NAMPT)-driven NAD^+^ biosynthesis sustains SIRT1 deacetylase activity, leading to deacetylation-dependent stabilization of PD-L1 in tumor cells [31,32]. Although direct validation of this axis in thyroid cancer is limited, thyroid malignancies, including ATC, have been reported to exhibit elevated NAMPT expression, and ATC also shows the highest PD-L1 staining among thyroid cancer subtypes, providing an indirect rationale for testing NAMPT or SIRT1 inhibitors as PD-L1 modulators in ATC [71,125]. SIRT1 also promotes Treg differentiation and immunosuppressive cytokine production by CD4^+^ T cells in other tumor contexts [130,131], mechanisms that may contribute to thyroid cancer, given the well-documented Treg infiltration in advanced disease [132,133].

SIRT6 has emerged as an important contributor to the formation and function of regulatory T cells. SIRT6 supports Treg metabolic fitness and FOXP3 stability, thereby reinforcing the immunosuppressive niche [31,32]. In addition, SIRT6-driven NK cell exhaustion has been reported in solid tumors [31], a finding that aligns with the limited NK cell cytotoxicity observed in advanced PTC and ATC [134]. The convergence of SIRT6’s tumor-cell-intrinsic oncogenic functions (Section 3.1 and Section 4) and its immunosuppressive effects in the TME makes SIRT6 a logical target for combination therapy, as a single SIRT6 inhibitor could simultaneously attenuate tumor proliferation, EMT, and immune evasion [26,28,32].

### 5.3. Immune-Activating Sirtuin Axes (SIRT2)

SIRT2 has emerged as a T cell metabolic checkpoint rather than a straightforward immune-activating sirtuin. In tumor-reactive T cells, SIRT2 activity can restrain metabolic fitness and effector function by regulating enzymes involved in glycolysis, oxidative phosphorylation, fatty acid oxidation, and glutaminolysis [129]. Consistent with this model, SIRT2-deficient T cells show enhanced metabolic activity, proliferation, and effector function, and pharmacologic SIRT2 inhibition improves the metabolic fitness and effector capacity of human tumor-infiltrating lymphocytes [129]. Therefore, available T-cell data support SIRT2 inhibition, rather than SIRT2 activation, as a potential strategy to augment antitumor immunity. Whether this approach can potentiate anti-PD-1 or other ICI-based therapies in thyroid cancer remains untested and should be evaluated in thyroid cancer-specific immune-competent or co-culture models [15,16,31,129].

Beyond T cells, SIRT2 has also been implicated in macrophage polarization, although the direction and therapeutic relevance of this effect may vary by tumor context [135,136]. The relevance of SIRT2-dependent immune regulation to thyroid cancer remains unexplored but warrants direct evaluation, particularly in *BRAF* V600E-mutant PTC, where immune checkpoint inhibitor monotherapy currently shows limited activity [16]. Rather than SIRT2 activation, selective SIRT2 inhibition warrants evaluation as a potential strategy to enhance tumor-reactive CD8^+^ T cell fitness and to improve the activity of pembrolizumab, spartalizumab, or other ICIs in thyroid cancer models, especially in ATC, where immune checkpoint blockade has shown partial activity [15,31,129,137].

### 5.4. Context-Dependent SIRT7 in the Immune Compartment

SIRT7 exhibits particularly complex immune regulation, exemplifying the context-dependent nature of sirtuin biology. In some tumor contexts, SIRT7 increases PD-L1-mediated immune evasion through an IRE1α–XBP1 endoplasmic reticulum stress response axis that drives PD-L1 transcription [32,138], while in other contexts, SIRT7 deacetylation of MEF2D reduces PD-L1 transcription, producing the opposite immunological outcome [31,32]. SIRT7-driven T cell metabolic fitness has also been described, supporting cytotoxic activity through enhanced ribosomal biogenesis [32,41]. The net effect of SIRT7 modulation on antitumor immunity in thyroid cancer is therefore highly context-dependent and requires direct experimental evaluation across PTC, FTC, and ATC subtypes.

A SIRT7-selective inhibitor may suppress tumor cell-intrinsic oncogenic activity driven by KIF23 and LATS1 desuccinylation. Its effects on antitumor immunity, however, are difficult to predict and require direct evaluation in thyroid cancer immune models. The substrate-class-selective inhibitors discussed in Section 7.3—which would block oncogenic desuccinylation while sparing housekeeping deacetylation—could in principle produce selective tumor cell killing without disrupting immune cell function, though this attractive concept remains to be experimentally validated [22,37].

### 5.5. Implications for Thyroid Cancer Immunotherapy

Despite accumulating mechanistic evidence, direct evidence for SIRT-mediated immune regulation specifically in thyroid cancer remains limited, with most current understanding extrapolated from other solid tumors [31,32]. This represents an actionable research priority. Several testable hypotheses warrant preclinical evaluation: SIRT1 or SIRT6 inhibition may help remodel immune-cold thyroid tumors by affecting PD-L1 regulation, regulatory T cell biology, or other immunosuppressive features; SIRT2 inhibition may enhance the metabolic fitness and effector function of tumor-reactive CD8^+^ T cells and thereby improve responsiveness to anti-PD-1-based therapy; and integrated SIRT expression profiles may serve as candidate biomarkers for immune checkpoint inhibitor response [15,16,31,32,129]. Combination strategies linking these immunological insights to current thyroid cancer immunotherapy regimens are discussed further in Section 8.

## 6. Sirtuin–Ferroptosis Axis and RAI-Refractory Disease

**Figure 5:**
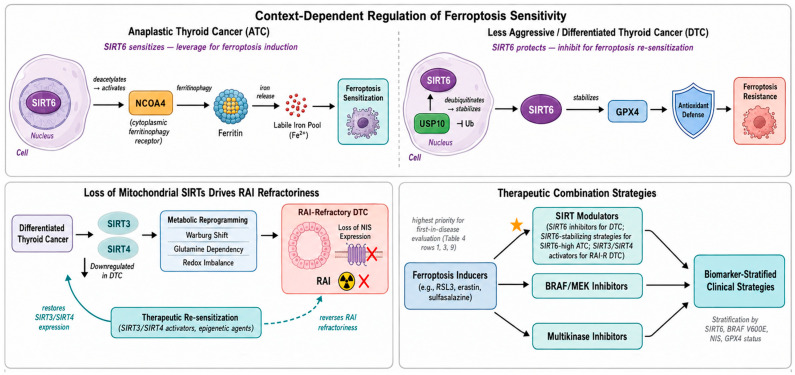
SIRT–ferroptosis axis and RAI-refractory thyroid cancer. **Top panel**: SIRT6 acts as a context-dependent regulator of ferroptosis sensitivity, playing opposite roles in different thyroid cancer subtypes. In anaplastic thyroid cancer (ATC, **left**), SIRT6 deacetylates and activates NCOA4 (a cytoplasmic ferritinophagy receptor), promoting NCOA4-dependent ferritinophagy, increasing the labile iron pool, and sensitizing cells to ferroptosis inducers (RSL3, erastin, sulfasalazine)—making SIRT6 a therapeutic asset to leverage for ferroptosis induction. In contrast, in differentiated thyroid cancer (DTC, **right**), USP10 deubiquitinates and stabilizes SIRT6, and the USP10–SIRT6–GPX4 axis stabilizes glutathione peroxidase 4 (GPX4), enhancing antioxidant defense and conferring ferroptosis resistance—making SIRT6 a target to inhibit for ferroptosis re-sensitization. This disease-state-specific dual role mandates context-tailored therapeutic strategies. **Bottom-left panel**: Downregulation of mitochondrial sirtuins SIRT3 and SIRT4 in DTC (semi-transparent ellipses, ↓ symbols) drives metabolic reprogramming (Warburg shift, glutamine dependency, redox imbalance), contributing to loss of NIS expression and the dedifferentiation phenotype underlying RAI refractoriness; SIRT3/SIRT4 activators and epigenetic agents are shown as a candidate re-sensitization strategy. **Bottom-right panel**: Therapeutic combinations integrating SIRT modulators (SIRT6 inhibitors for DTC; SIRT6-stabilizing strategies for SIRT6-high ATC; SIRT3/SIRT4 activators for RAI-refractory DTC) with ferroptosis inducers, BRAF/MEK inhibitors, or multikinase inhibitors enable subtype-specific strategies. The combination of ferroptosis inducers with SIRT modulators is the highest priority for biomarker-driven preclinical validation. NCOA4, nuclear receptor coactivator 4; USP10, ubiquitin-specific peptidase 10; GPX4, glutathione peroxidase 4; NIS, sodium-iodide symporter; RAI, radioactive iodine. Node colors follow the scheme used throughout the figures: purple = tumor-promoting sirtuins; teal = tumor-suppressive sirtuins; coral = context-dependent; grey = underexplored. Arrows: → activation/stabilization; ↓ downregulation; dashed arrow = hypothesized or not-yet-validated relationship; ★ highest priority.

Ferroptosis—an iron-dependent form of regulated cell death driven by lipid peroxidation—has emerged as an attractive therapeutic vulnerability in thyroid cancer, particularly in ATC and potentially in dedifferentiated or RAI-refractory DTC [33,34,35,139,140]. However, the strength of evidence differs by disease context. In ATC, SIRT6-driven NCOA4-dependent ferritinophagy has direct preclinical support as a mechanism that increases ferroptosis sensitivity [35]. In contrast, the relevance of this SIRT6–ferroptosis axis to RAI-refractory DTC remains largely inferential and should be tested in dedicated dedifferentiated and RAI-refractory models [34]. Figure 5 integrates the SIRT–ferroptosis axis with the dedifferentiation phenotype that defines RAI-refractory disease.

### 6.1. Ferroptosis Biology in Thyroid Cancer

Ferroptosis is driven by the accumulation of lipid peroxides through the action of polyunsaturated fatty acid-containing phospholipids and labile iron, with glutathione peroxidase 4 (GPX4) and the cystine/glutamate antiporter system Xc^−^, encoded by SLC7A11, representing the principal cellular defenses [140,141]. Thyroid cancer cells, particularly those of dedifferentiated and anaplastic histology, exhibit altered iron homeostasis (increased ferritin storage and ferritinophagy capacity), elevated polyunsaturated fatty acid incorporation via ACSL4 and LPCAT3, and dependence on antioxidant defenses driven by oncogenic BRAF, RAS, and PIK3CA mutations [33,34,142]. This metabolic configuration creates a therapeutic window for ferroptosis induction that is particularly pronounced in ATC, where redox imbalance is severe and conventional therapeutic options are limited [7,33].

The key ferroptosis regulators in thyroid cancer extend beyond GPX4 and SLC7A11 to include heme oxygenase 1 (HO-1) for iron homeostasis, the autophagy receptor NCOA4 for ferritinophagy [7,143], and the recently identified GPR34–USP8 axis that suppresses ferroptosis in ATC [33]. m^6^A RNA modification regulators (ALKBH5, FTO) and several non-coding RNAs have also been shown to modulate ferroptosis sensitivity in thyroid cancer cell lines [33,34].

### 6.2. SIRT6 as a Dual Regulator of Ferroptosis Sensitivity

It should be noted that the SIRT6–NCOA4 ferritinophagy axis in ATC is currently supported by a single primary study [35] and, although it includes in vivo xenograft data, awaits independent replication; this is reflected in the evidence-level designation in Table 2. SIRT6 has been identified as a potent regulator of ferroptosis sensitivity in ATC, although its directionality may vary across thyroid cancer contexts [33,34]. In ATC cell models, SIRT6 overexpression sensitized cells to ferroptosis inducers, including RSL3, erastin, ML210, and ML162, whereas SIRT6 knockout conferred resistance to ferroptosis induction [35]. Mechanistically, SIRT6 promoted NCOA4-dependent autophagic degradation of ferritin, increased intracellular Fe^2+^ availability, and thereby enhanced lipid peroxidation-driven ferroptotic cell death [35,67]. In vivo, sulfasalazine, a system Xc^−^ inhibitor, showed therapeutic activity against SIRT6-upregulated thyroid cancer xenografts, supporting SIRT6-high ATC as a potential context for ferroptosis-inducing strategies [35,144].

In contrast, in less aggressive thyroid cancer contexts, the USP10–SIRT6–GPX4 axis has been reported to suppress ferroptosis: USP10 deubiquitinates and stabilizes SIRT6, which in turn facilitates expression of the ferroptosis suppressor GPX4 and protects thyroid cancer cells from erastin-induced lipid peroxidation, with concomitant reductions in intracellular Fe^2+^, malondialdehyde, lipid ROS, and mitochondrial superoxide; loss of USP10 represses thyroid cancer xenograft growth in vivo [33,34,145].

The apparent paradox of SIRT6 acting as both a ferroptosis sensitizer (in ATC) and a ferroptosis protector (in DTC) likely reflects context-dependent dominance of two competing molecular axes that operate within the same enzymatic activity profile. First, the NCOA4–ferritinophagy axis, in which SIRT6-mediated deacetylation potentiates NCOA4-driven autophagic degradation of ferritin and consequent expansion of the labile iron pool, predominates in metabolically aggressive ATC. In this context, iron release exceeds GPX4’s antioxidant buffering capacity, leading to lipid peroxidation-driven ferroptotic cell death [35,67]. Second, the USP10–SIRT6–GPX4 axis, in which deubiquitination-mediated SIRT6 stabilization sustains GPX4 expression and antioxidant defense, predominates in less aggressive DTC contexts where iron flux is modest and GPX4-mediated peroxide reduction is sufficient to suppress lipid peroxidation [145]. Several factors likely determine which axis is dominant in a given tumor: (i) tumor cell mutational background, with TP53 loss-of-function and *BRAF* V600E in ATC favoring metabolic reprogramming that shifts iron homeostasis toward ferritinophagy [33,115]; (ii) baseline NCOA4 and GPX4 expression levels, which set the threshold ratio of pro-ferroptotic versus anti-ferroptotic flux [35]; (iii) the strength and class of incoming ferroptotic stimuli, with system Xc^−^ inhibition (sulfasalazine, erastin) more effectively engaging the ferritinophagy-dominant ATC state and GPX4-targeting inducers (RSL3) being potentially active in both states [35,144]; and (iv) cellular redox capacity, including NADPH availability and FSP1/CoQ10 status, which modulates the buffering effect of GPX4-independent pathways [33]. This biomarker-stratified framework—rather than a single SIRT6-targeting paradigm—should guide preclinical validation and translational design and is further developed in Section 8.4 (combination strategies for SIRT6-high ATC) and Section 10 (biomarker considerations for patient selection).

The therapeutic implication of this dichotomy is striking: SIRT6-overexpressing ATC tumors are predicted to be hypersensitive to ferroptosis inducers, providing a rationale for combining sulfasalazine, RSL3-derivative agents, or anlotinib with SIRT6-stabilizing strategies in ATC [33], while SIRT6 inhibition may be the appropriate strategy in DTC contexts where the USP10–SIRT6–GPX4 axis confers ferroptosis resistance and tumor survival benefit.

In summary, SIRT6 modulation should be guided by disease state and underlying biology: (i) in *BRAF* V600E-mutant PTC and aggressive PTC where SIRT6 amplifies MAPK signaling, HIF-1α/EMT, and Warburg-like metabolism, SIRT6 inhibition (e.g., OSS_128167) is the rational strategy to be combined with BRAF/MEK inhibitors or multikinase inhibitors; (ii) in SIRT6-high ATC where SIRT6 promotes NCOA4-dependent ferritinophagy, preserving or leveraging SIRT6 activity together with ferroptosis inducers (sulfasalazine, RSL3 derivatives) is the rational strategy, since SIRT6 inhibition in this context would attenuate the ferroptosis vulnerability; and (iii) in DTC contexts where the USP10–SIRT6–GPX4 axis confers ferroptosis resistance, SIRT6 inhibition is again favored. This disease-state-specific decision logic, rather than a single SIRT6-targeting paradigm, should guide preclinical and translational design.

### 6.3. Connection to RAI-Refractory Disease

RAI refractoriness in DTC arises from loss of thyroid-specific differentiation, particularly downregulation of the sodium-iodide symporter (NIS, encoded by SLC5A5), and represents one of the most pressing unmet needs in thyroid oncology [8,146,147]. Recent literature has linked dedifferentiation, metabolic reprogramming, and ferroptosis vulnerability as interconnected features of advanced thyroid cancer [33,34,115]. In particular, dedifferentiated thyroid tumors exhibit Warburg-like metabolism, increased dependence on glutamine for biosynthesis, and altered redox homeostasis—all processes regulated by mitochondrial sirtuins (Section 4.5) [29,74].

The convergence of dedifferentiation, metabolic reprogramming, and ferroptosis sensitivity suggests that SIRT-targeted strategies may complement RAI-directed therapy, but they should not yet be presented as established RAI-resensitization agents [29,34,115,147]. Restoration of SIRT3 or SIRT4 activity may help reverse metabolic features associated with dedifferentiation, including mitochondrial dysfunction, oxidative stress, glycolytic shift, and altered glutamine metabolism [29,74]. However, direct evidence that SIRT3 or SIRT4 restoration restores NIS expression or increases RAI uptake in thyroid cancer models is currently lacking. This concept should therefore be framed as a testable redifferentiation hypothesis rather than an established RAI-resensitization mechanism [146,147,148]. In parallel, induction of ferroptosis through SIRT6-associated ferritinophagy may provide an alternative cell-killing strategy in tumors that have escaped RAI-mediated cytotoxicity, although this should be distinguished from true restoration of iodine avidity [33,34].

### 6.4. Combination Potential: Ferroptosis Inducers and SIRT Modulators

The integration of SIRT modulators with ferroptosis-inducing agents represents a promising but still largely preclinical combination strategy for advanced thyroid cancer. Preclinical data support the broader concept that therapy-resistant thyroid cancer states may be vulnerable to GPX4 blockade and ferroptosis induction [149,150], while ATC-focused evidence suggests that SIRT6-high status may define a subset with increased susceptibility to ferroptosis-inducing strategies [33,35]. However, combination strategies incorporating SIRT6-directed modulation with BRAF/MEK inhibition or ferroptosis induction should first undergo biomarker-driven mechanistic and preclinical validation before early-phase clinical testing is considered.

First, in SIRT6-high ATC models, sulfasalazine or other system Xc^−^-targeting approaches should be tested for cooperation with SIRT6-driven NCOA4-dependent ferritinophagy [35,144,151]. Second, anlotinib, a multikinase inhibitor with reported ferroptosis-inducing activity, may be evaluated with SIRT-directed strategies in RAI-refractory DTC models, but this remains hypothesis-generating and requires disease-specific validation [34,152]. Third, natural compound ferroptosis inducers, including vitamin C, neferine, curcumin, and shikonin, may serve as exploratory combination partners in ATC models, although their potency, pharmacokinetics, and SIRT6-dependence require further characterization [33,34]. Fourth, combinations of dabrafenib plus trametinib with ferroptosis inducers and SIRT6-directed strategies should be prioritized for mechanistic testing in *BRAF* V600E-mutant, biomarker-selected ATC models rather than immediate clinical evaluation [9,10,149].

The full clinical translation pathway, including detailed combination trial designs, is developed in Section 8, while the biomarker considerations needed to identify patients most likely to benefit from these regimens—including SIRT6 expression, GPX4 levels, ferritinophagy capacity, and lipid metabolism profiling—are outlined in Section 10.

## 7. Sirtuin-Targeted Therapeutics: From Bench to Bedside

The dichotomous role of sirtuins in thyroid cancer creates a uniquely complex therapeutic landscape: tumor-promoter sirtuins (SIRT1, SIRT6, SIRT7) require selective inhibitors, while tumor-suppressor sirtuins (SIRT3, SIRT4) would benefit from selective activators or strategies that restore their lost expression [18,37,46]. This section reviews the current pharmacological toolkit for SIRT modulation, summarizes the available preclinical evidence in thyroid cancer models, and outlines the substantial clinical translation gap that defines this field. Table 3 provides a comprehensive summary of SIRT modulators with thyroid cancer relevance, including their primary targets, chemical class, and the strength of preclinical evidence. It is important to emphasize that the great majority of SIRT modulators currently available—including OSS_128167 (SIRT6), EX-527 (SIRT1), and AGK2/AK-7 (SIRT2)—are research tool compounds rather than clinically developed drugs, and that bona fide selective SIRT3, SIRT4, and SIRT7 modulators with drug-like properties do not yet exist. None of these compounds has thyroid cancer-specific in vivo efficacy data, with the partial exception of SIRT6 inhibition in the NCOA4-ferritinophagy ATC xenograft model [35]. Table 4 therefore includes an explicit “translation readiness” column to prevent any impression that these agents are near-term clinical options in thyroid cancer.

### 7.1. Sirtuin Activators (STACs)

Sirtuin-activating compounds (STACs) increase sirtuin enzymatic activity and have been developed primarily to mimic the metabolic and longevity-promoting effects of caloric restriction [169,170]. The natural polyphenol resveratrol was the first identified STAC and remains the most extensively studied; it allosterically activates SIRT1 by lowering the K_m_ for both NAD^+^ and acetylated substrates [169,171]. In thyroid cancer, resveratrol has shown anti-proliferative effects in PTC cell lines (B-CPAP, K1, TPC-1) at micromolar concentrations [153,172], but its biological effects are pleiotropic and likely involve multiple targets beyond SIRT1, including AMPK activation, NF-κB inhibition, and direct antioxidant effects [37,173]. Importantly, in the *Pten*-null mouse model of thyroid cancer, resveratrol accelerated rather than delayed tumor progression [23], underscoring that SIRT1 activation may be undesirable in PTEN-deficient thyroid cancer and highlighting the importance of patient stratification for any STAC-based intervention.

Synthetic STACs developed by Sirtris Pharmaceuticals (now part of GSK)—including SRT1720, SRT2104, and SRT2379—exhibit greater potency and selectivity for SIRT1 than resveratrol [171,174]. SRT2104 has progressed through phase I and phase II trials in metabolic and inflammatory diseases and has an acceptable safety profile [154,155], but has not been tested in thyroid cancer. Given SIRT1’s tumor-promoting role in PTEN-deficient thyroid cancer [23], SIRT1 activators are unlikely to be clinically useful in this context. STACs may instead find use in a fundamentally different setting: as selective activators of tumor-suppressor sirtuins SIRT3 and SIRT4, where activation would be therapeutically desirable [29,46].

To date, isoform-selective SIRT3 and SIRT4 activators with drug-like properties remain elusive, although several patents and early-stage compounds have been disclosed in the field [37,46]. Honokiol, a biphenolic natural product, has been reported to enhance SIRT3 activity in cardiac and metabolic contexts [158] and shows preliminary antitumor activity in some cancer models, although its multi-target profile complicates the interpretation of SIRT3-specific effects [157]. Adjudin and dihydrorotenone analogs have been proposed as scaffolds for SIRT4-targeting compounds, exploiting SIRT4’s unique substrate-binding pocket [37]. The development of bona fide SIRT3 and SIRT4 activators represents a major medicinal chemistry challenge and an opportunity for thyroid cancer-specific drug development, particularly for ATC, where loss of mitochondrial sirtuin function contributes to dedifferentiation [29,74]. Strategies that restore expression of these tumor-suppressor sirtuins—for example, through epigenetic agents (HDAC inhibitors, DNA methyltransferase inhibitors) that reverse promoter silencing—represent a complementary strategy that warrants preclinical evaluation [86,175].

### 7.2. Sirtuin Inhibitors (STICs)

Given that SIRT1, SIRT6, and SIRT7 act as tumor promoters in most thyroid cancer contexts (Section 3), sirtuin inhibitors (STICs) represent the more directly translatable therapeutic approach. The current preclinical inhibitor toolkit includes compounds with varying isoform selectivity and mechanistic profiles [22,37].

SIRT1 inhibitors. EX-527 (selisistat) is the most clinically advanced SIRT1 inhibitor, having completed phase II trials in Huntington’s disease, where it demonstrated good tolerability [160,176]. EX-527 is a competitive inhibitor that traps SIRT1 in a deacetylation-incompetent state [177,178]. In thyroid cancer cell lines, EX-527 reverses SIRT1-mediated chemoresistance to etoposide [73] and reduces proliferation in PTEN-deficient backgrounds, though in vivo validation in thyroid cancer xenografts is limited. Sirtinol is an older, less selective SIRT1/SIRT2 inhibitor that has been used as a tool compound in thyroid cancer studies [161,179]. Cambinol [162] and inauhzin [163] represent additional SIRT1 inhibitors with preclinical activity in various cancer models, though thyroid-specific data are sparse.

SIRT2 inhibitors. AGK2 is a brain-permeable SIRT2-selective inhibitor used primarily in neurodegenerative disease research [164,180], and AK-7 is a closely related compound with improved pharmacokinetics [165]. Despite the underexplored role of SIRT2 in thyroid cancer, the availability of these well-characterized tool compounds makes SIRT2 a tractable target for early thyroid cancer drug discovery efforts [37]. In the immunotherapy context, these compounds may be useful as tool inhibitors to test whether SIRT2 blockade enhances tumor-reactive T-cell metabolic fitness and effector function in thyroid cancer models, as suggested by non-thyroid tumor-reactive T-cell studies [129].

SIRT3 inhibitors. Because SIRT3 functions as a tumor suppressor in thyroid cancer [30,86], the therapeutic inhibition of SIRT3 is contraindicated. However, isoform-selective SIRT3 inhibitors such as 3-TYP are useful as research tools to validate SIRT3-dependent phenotypes [166,181].

SIRT6 inhibitors and activators. Notably, SIRT6 displays pronounced context dependence, functioning either as a tumor promoter in *BRAF* V600E-mutant PTC and ATC—primarily through the BRAF/ERK/Mcl-1 and HIF-1α/EMT axes [26,28]—or as a tumor suppressor in other malignancies and in the maintenance of normal tissue homeostasis [68,182]. The pyrrolopyrimidine compound OSS_128167 is a SIRT6-selective inhibitor that has shown preclinical activity in multiple myeloma and pancreatic cancer models [103,104] and is a leading candidate for thyroid cancer-focused evaluation given the prominence of SIRT6 in BRAF-driven PTC. Conversely, SIRT6 activators such as MDL-800 and MDL-801 have been developed [159,183] and could theoretically be useful in tissue contexts where SIRT6 acts as a tumor suppressor; their application in thyroid cancer would require careful subtype-specific evaluation.

SIRT7 inhibitors. SIRT7 inhibitor development is at an earlier stage than for other sirtuins, reflecting the relatively recent recognition of SIRT7 as a major oncogenic player. 9,7491 and a series of imidazothiazole derivatives have been reported as preliminary SIRT7 inhibitors with low micromolar potency [37,184]. Given the central role of SIRT7 in non-canonical PTM regulation in thyroid cancer (KIF23 in ATC, LATS1 in PTC; [25,27]) (Section 3.3), SIRT7-selective inhibitors represent perhaps the most exciting near-term opportunity for thyroid cancer-specific drug development. Structure-based design exploiting the unique substrate-binding groove of SIRT7 may enable the rational development of compounds with both potency and isoform selectivity [22,185].

Pan-SIRT inhibitors. Nicotinamide is the endogenous pan-SIRT inhibitor (the product of the deacetylation reaction) and has been evaluated for chemoprevention of non-melanoma skin cancer [167]. MC2494 is a synthetic pan-SIRT inhibitor that has shown anti-proliferative effects in multiple cancer cell lines [168,186]. Although pan-SIRT inhibition is conceptually problematic given the dichotomous roles of different SIRTs in thyroid cancer, these compounds remain useful tool compounds and may inform isoform-selective drug development.

### 7.3. Drug Discovery Trends (2024–2025)

Several trends have emerged in the field of SIRT modulators over the past two years that are particularly relevant to thyroid cancer drug development. First, structure-based design enabled by high-resolution sirtuin crystal structures has accelerated the development of isoform-selective inhibitors, with multiple recent publications reporting novel scaffolds for SIRT2 and SIRT6 [22,37,185]. Cryo-EM structures of SIRT7 in complex with substrate peptides, published in 2024, are particularly notable for providing detailed views of nucleosome-bound deacylase active sites and for already enabling the rational design of preliminary SIRT7-selective inhibitors [187].

Second, proteolysis-targeting chimera (PROTAC) approaches are being applied to sirtuins, with early reports of SIRT1 and SIRT2 degraders that achieve greater target depletion than catalytic inhibition alone [37,188]—a strategy that may be particularly useful for sirtuins with scaffolding functions independent of enzymatic activity. PROTAC-mediated SIRT7 degradation, in particular, could overcome the redundancy between SIRT7 deacetylase and desuccinylase activities and is an active area of investigation [22].

Third, fragment-based drug discovery and AI-assisted design have begun to yield compounds with novel mechanisms of action, including allosteric modulators that bias sirtuin activity toward specific substrate classes (for example, favoring desuccinylation over deacetylation) [37]. Computational platforms trained on sirtuin substrate–enzyme interactions can now predict selective binders for individual SIRT isoforms with reasonable hit rates, accelerating early-stage drug discovery [22].

Fourth, recognition of non-canonical sirtuin activities (desuccinylation, demalonylation, deglutarylation) has motivated the development of activity-specific assays and the search for substrate-class-selective inhibitors that could spare housekeeping deacetylase function while blocking oncogenic desuccinylase activity—a particularly attractive concept for SIRT7 in thyroid cancer [22,25,27]. Such substrate-class-selective inhibition would represent a significant therapeutic advance, allowing intervention on the oncogenic SIRT7-KIF23 and SIRT7-LATS1 axes [25,27] while preserving SIRT7’s physiological deacetylase functions in ribosomal biogenesis and other housekeeping processes [41,63].

### 7.4. Clinical Trial Landscape and the Gap in Thyroid Cancer

Despite over a decade of preclinical development, the clinical translation of SIRT modulators in oncology remains in its infancy. A search of ClinicalTrials.gov (search date: January 2026; query terms: “sirtuin” OR “SIRT1” OR “SIRT2” OR “SIRT3” OR “SIRT6” OR “SIRT7” AND “cancer”) revealed fewer than ten active or completed oncology trials of SIRT-targeted agents, with most evaluating resveratrol or its analogs as adjuncts to standard chemotherapy in colorectal, breast, or hematologic malignancies [22,37]. No clinical trial has specifically evaluated a SIRT modulator in thyroid cancer, despite the substantial preclinical evidence reviewed above and the urgent unmet need in BRAF-refractory PTC and ATC [7,9].

This gap reflects several converging challenges: the absence of validated predictive biomarkers, the complexity of subtype- and mutation-specific SIRT biology, the relative rarity of the most therapy-relevant subtypes (RAI-refractory DTC, ATC), and the limited commercial incentive for thyroid-specific drug development relative to more common cancers. Nonetheless, the convergence of biological insight (particularly the 2024–2025 desuccinylation findings), clinical need, and improving inhibitor pharmacology suggests that the next five years could see the first thyroid cancer-focused clinical trials of SIRT modulators [22,25,27], most likely as combination partners with established BRAF/MEK inhibitors or multikinase inhibitors. The combination strategies discussed in Section 8 outline the most promising trial designs, and the biomarker considerations addressed in Section 10 highlight the patient selection frameworks that will determine the success or failure of these efforts.

Given the rarity of the most therapy-relevant subtypes (RAI-refractory DTC, *BRAF* V600E-mutant ATC), a thyroid cancer-specific basket trial design—enrolling biomarker-defined cohorts (SIRT6-high, *BRAF* V600E, SIRT7-high with KIF23/LATS1 succinylation status) across PTC, FTC, and ATC histologies and stratifying by SIRT expression profile—would be a practical first step. Once the preclinical prerequisites outlined in Section 10.3 have been met, a multi-center, investigator-initiated phase I/II umbrella trial pairing OSS_128167 or EX-527 with standard-of-care backbones (dabrafenib + trametinib for *BRAF* V600E disease; lenvatinib for RAI-refractory DTC) and incorporating mandatory pre- and on-treatment biopsies for pharmacodynamic biomarker assessment would be the most efficient way to generate the first clinical data for this target class in thyroid cancer.

## 8. Combination Strategies and Clinical Translation

**Figure 6:**
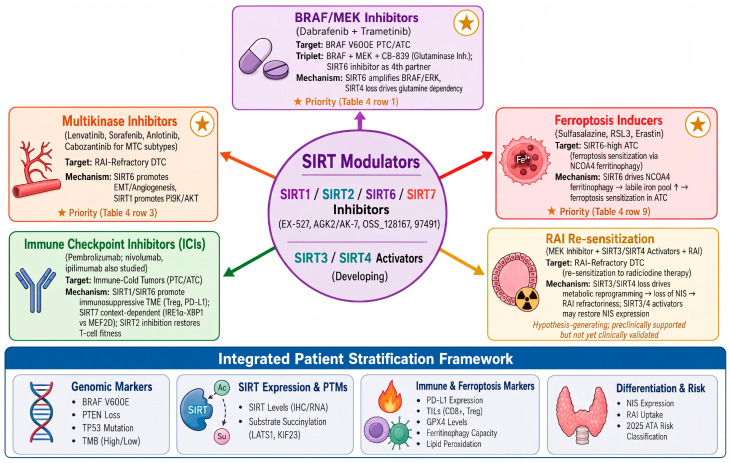
SIRT modulator combination strategies in thyroid cancer—integrated clinical translation framework. SIRT modulators (central hub)—including SIRT1/SIRT2/SIRT6/SIRT7 inhibitors (EX-527, AGK2/AK-7, OSS_128167, 97491 series) and SIRT3/SIRT4 activators (in development)—serve as combination partners with five established or emerging therapeutic axes. Top (12 o’clock): BRAF/MEK inhibitor combinations (Section 8.1) for *BRAF* V600E-mutant disease, including triplet integration (BRAF + MEK + CB-839 glutaminase inhibitor) with SIRT6 inhibitors as an additional partner. Upper-left (10 o’clock): multikinase inhibitor combinations (Section 8.2) for RAI-refractory differentiated thyroid cancer, leveraging lenvatinib, sorafenib, anlotinib, or cabozantinib (for medullary subtypes). Lower-left (8 o’clock): immune checkpoint inhibitor combinations (Section 8.3) for immune-cold tumors (mechanism: SIRT1/SIRT6 promote immunosuppressive TME; SIRT7 context-dependent; SIRT2 inhibition restores T-cell metabolic fitness). Upper-right (2 o’clock): ferroptosis inducer combinations (Section 8.4) for SIRT6-overexpressing anaplastic thyroid cancer. Lower-right (4 o’clock, new): RAI re-sensitization (Section 8.5) integrating MEK inhibitors with SIRT3/SIRT4 activators to restore NIS expression in RAI-refractory DTC (hypothesis-generating; preclinically supported but not yet clinically validated). Three combinations are designated the highest priority for biomarker-driven preclinical validation (★, marked on BRAF/MEK, multikinase, and ferroptosis boxes; corresponding to Table 4 rows 1, 3, and 9, respectively). The bottom band illustrates the integrated patient stratification framework spanning genomic markers (*BRAF* V600E, PTEN, TP53, TMB), SIRT expression profiles (including substrate-specific PTM markers such as LATS1 and KIF23 succinylation), immune markers, ferroptosis susceptibility markers, and differentiation markers, including 2025 ATA guideline-based risk classification [36]. Successful clinical translation requires biomarker-guided patient selection across all five combination strategies. ICI, immune checkpoint inhibitor; RAI, radioactive iodine; PTM, post-translational modification; ATA, American Thyroid Association; NIS, sodium-iodide symporter; TMB, tumor mutational burden; TILs, tumor-infiltrating lymphocytes. SIRT modulator color coding (hub): SIRT1/SIRT6 in purple, SIRT2 in teal, SIRT3/SIRT4 in teal, SIRT7 in coral—consistent with Figure 1, Figure 3, Figure 4 and Figure 5.

The mechanistic and therapeutic insights developed in the preceding sections converge on a unifying translational hypothesis: SIRT modulators are most likely to provide meaningful therapeutic benefit when combined with established thyroid cancer therapies, rather than as monotherapies. This section outlines the most promising combination strategies, organized by the principal therapeutic backbone, and identifies the patient populations and biomarker frameworks that should guide biomarker-driven preclinical validation and, where preclinical efficacy and safety are subsequently established, eventual clinical evaluation. Throughout this section and in Table 4, each strategy is labeled by evidence level—thyroid cancer-specific in vivo evidence, thyroid cancer-specific in vitro evidence, evidence from other cancer types, or conceptual rationale—to make the current maturity of each approach explicit. Figure 6 integrates these combination strategies, and Table 4 summarizes the rationale, supporting evidence, and developmental status of each combination.

Among the fourteen combinations summarized in Table 4, three are designated as the highest priority for biomarker-driven preclinical validation (rows 1, 3, and 9). Row 1 (OSS_128167 + dabrafenib/trametinib in *BRAF* V600E PTC and ATC) is prioritized because the SIRT6–BRAF/ERK/Mcl-1 axis has the strongest thyroid cancer-specific preclinical support (Table 2; thyroid cancer-specific in vitro and in vivo evidence) and addresses MAPK reactivation, the principal resistance mechanism to BRAF/MEK blockade. Row 3 (OSS_128167 + lenvatinib or sorafenib in RAI-refractory DTC) addresses the largest patient population with unmet need and leverages SIRT6 inhibition of HIF-1α/EMT and angiogenesis, which is mechanistically complementary to multikinase inhibitor anti-angiogenic activity (thyroid cancer-specific in vitro evidence; in vivo combination data not yet available). Row 9 (SIRT6-stabilizing strategies + sulfasalazine in SIRT6-high ATC) is prioritized because the SIRT6–NCOA4 ferritinophagy axis is directly supported by in vivo ATC xenograft data (thyroid cancer-specific in vivo evidence) [35] and offers a fundamentally distinct mechanism of action for the most lethal thyroid cancer subtype. These three combinations are prioritized for rigorous biomarker-driven preclinical validation—including patient-derived xenograft and organoid studies with defined pharmacodynamic endpoints—as a necessary precondition for any subsequent clinical evaluation. These three combinations are marked in Table 4 and Figure 6 with a star (★).

### 8.1. Combinations with BRAF/MEK Inhibitors

The most clinically advanced opportunity lies in combining BRAF/MEK inhibitor therapy (dabrafenib plus trametinib) with treatment strategies for *BRAF* V600E–mutant thyroid cancer, including PTC and the substantial subset of ATC that harbors this mutation [6,9,10]. The mechanistic rationale is multilayered: SIRT6 amplifies BRAF/ERK output through Mcl-1 stabilization (Section 4.1) [26], SIRT1 cooperates with PTEN loss to sustain parallel PI3K/AKT signaling (Section 4.2) [23], and SIRT4 loss enables glutamine-dependent metabolic adaptation (Section 4.5) [21,74]. A SIRT6 inhibitor (e.g., OSS_128167; [104]) added to dabrafenib + trametinib could simultaneously attenuate downstream MAPK output and pre-empt the MAPK reactivation that drives BRAF inhibitor resistance [101,102], while SIRT4 reconstitution or glutaminase inhibition (CB-839/telaglenastat; [117]) could close the metabolic escape route. Such triplet regimens warrant rigorous preclinical validation in thyroid cancer models, including pharmacological feasibility and safety assessment, before clinical evaluation can be considered in *BRAF* V600E-mutant ATC, where the unmet need is most acute [7,11].

### 8.2. Combinations with Multikinase Inhibitors

For RAI-refractory DTC, the established multikinase inhibitors lenvatinib and sorafenib provide the current standard of care but are limited by toxicity (hypertension, diarrhea, fatigue) and finite duration of response (median progression-free survival of 12–18 months for lenvatinib) [12,13]. Adding SIRT modulators could enhance both efficacy and durability through complementary mechanisms. SIRT6 inhibitors could attenuate HIF-1α-driven EMT and angiogenesis (Section 4.3) [28,109], reinforcing the anti-angiogenic activity of multikinase inhibitors and potentially allowing dose reduction to mitigate toxicity. SIRT1 inhibitors (EX-527) [160] could disrupt the parallel PI3K/AKT axis that contributes to acquired resistance to vascular endothelial growth factor receptor blockade [23,73]. Anlotinib, which has intrinsic ferroptosis-inducing activity (Section 6.4) [34,152], is an especially attractive candidate for combination with SIRT-directed strategies designed to enhance ferroptosis sensitivity in dedifferentiated tumors. Cabozantinib, which was recently approved for DTC after lenvatinib failure [14], may likewise benefit from combination with SIRT inhibitors. However, direct preclinical evidence for this approach is currently lacking, making it an important priority for future research.

### 8.3. Combinations with Immune Checkpoint Inhibitors

The substantial fraction of advanced thyroid cancer patients who do not respond to ICI monotherapy represents a critical unmet need that SIRT modulators may help address (Section 5) [15,16]. Three combination concepts merit prospective evaluation. First, SIRT1 or SIRT6 inhibitors combined with pembrolizumab/spartalizumab in PD-L1-low or immune-cold tumors could disrupt the NAMPT-SIRT1-PD-L1 axis and SIRT6’s Treg-supportive functions, converting cold tumors into immunoreactive ones [31,32]. Second, selective SIRT2 inhibition combined with anti-PD-1 therapy could be explored as a strategy to enhance tumor-reactive CD8^+^ T cell metabolic fitness and effector function, although this approach has not yet been validated in thyroid cancer models [31,129]. Third, SIRT7-selective inhibitors plus ICI could simultaneously address tumor-cell-intrinsic SIRT7 oncogenic functions [25,27] and modulate immune checkpoint expression [32].

### 8.4. Combinations with Ferroptosis Inducers

The SIRT6–ferroptosis axis (Section 6.2) offers a mechanistically grounded yet largely preclinical combination opportunity, particularly in SIRT6-high ATC. Direct preclinical evidence indicates that SIRT6 can increase ferroptosis sensitivity in ATC by promoting NCOA4-dependent ferritinophagy, thereby increasing intracellular iron availability and lipid peroxidation [33,35,151]. In this context, sulfasalazine or other system Xc^−^-targeting approaches may cooperate with SIRT6-driven ferritinophagy to deplete antioxidant defenses and amplify ferroptotic cell death, with lipid peroxidation, labile iron, SIRT6 expression, and NCOA4 levels serving as potential pharmacodynamic biomarkers [35,146,151]. RSL3 derivatives or GPX4-targeting compounds could provide an alternative strategy for inducing ferroptosis in tumors in which system Xc^−^ inhibition is insufficient or bypassed through compensatory SLC7A11-dependent mechanisms [142,152]. Natural compound ferroptosis inducers, including vitamin C, neferine, curcumin, and shikonin, have shown preclinical activity in ATC-related ferroptosis models and may offer lower-toxicity candidates for combination testing, although their potency, pharmacokinetics, and SIRT6-dependence require further validation [33,34].

Importantly, SIRT6-related therapeutic logic should be disease-state specific. In *BRAF* V600E-mutant papillary thyroid cancer (PTC) or advanced thyroid cancer contexts where SIRT6 reinforces MAPK signaling, HIF-1α stabilization, epithelial–mesenchymal transition (EMT), or metabolic adaptation, SIRT6 inhibition may be explored with BRAF/MEK inhibitors [26,28]. By contrast, in SIRT6-high ATC, ferroptosis-inducing strategies may exploit rather than inhibit SIRT6-driven NCOA4-dependent ferritinophagy [33,35]. Therefore, adding SIRT6 modulation to dabrafenib plus trametinib and ferroptosis-inducing therapy should currently be considered a logical next step for mechanistic and preclinical evaluation in biomarker-selected ATC models, rather than a ready strategy for clinical evaluation. Patient selection should be guided by SIRT6 expression, NCOA4 levels, GPX4/SLC7A11 status, ferritinophagy capacity, and lipid peroxidation susceptibility profiling [33,34,35,151].

### 8.5. Potential RAI Re-Sensitization Strategies Involving Mitochondrial Sirtuins

Restoration of RAI sensitivity in dedifferentiated DTC remains a major therapeutic goal, particularly because RAI refractoriness is closely linked to loss of thyroid-specific differentiation and reduced NIS expression [8,146,147]. In this context, restoring mitochondrial sirtuin activity is a hypothesis-generating strategy rather than an established redifferentiation approach. SIRT3 activators or epigenetic agents that restore SIRT3/SIRT4 expression may counteract metabolic features associated with dedifferentiation, including mitochondrial dysfunction, oxidative stress, aerobic glycolysis, and glutamine-dependent metabolic adaptation [29,74]. However, whether these interventions can directly restore NIS expression, iodine uptake, or RAI sensitivity remains unknown. Therefore, combinations of mitochondrial sirtuin restoration with MAPK inhibitor-based redifferentiation, such as selumetinib priming followed by RAI, should first be evaluated in proof-of-concept preclinical models of RAI-refractory DTC before clinical translation is considered [146,147,148]. It must be stated explicitly that this RAI re-sensitization strategy currently rests on conceptual rationale rather than direct experimental evidence. While SIRT3 and SIRT4 downregulation are associated with the metabolic dedifferentiation that characterizes RAI-refractory disease, no published study has yet demonstrated that SIRT3 or SIRT4 restoration restores sodium-iodide symporter (NIS) expression, increases radioiodine uptake, or improves RAI response in thyroid cancer models. The proposed link between mitochondrial sirtuin restoration and redifferentiation (Figure 5 and Figure 6) is therefore a hypothesis-generating concept (conceptual rationale) that requires direct experimental validation—including NIS expression assays, radioiodine uptake studies, and functional redifferentiation endpoints—before it can be regarded as a viable therapeutic strategy.

Clinical translation of these combination strategies will likely depend on rigorous patient stratification based on SIRT expression profiles, mutation status (*BRAF* V600E, PTEN loss), and complementary biomarkers (PD-L1, GPX4, ferritinophagy capacity) [36,94,122]. Biomarker considerations and the broader clinical translation framework are developed further in Section 10.

## 9. Conclusions

This review synthesizes the rapidly evolving understanding of sirtuins (SIRT1–SIRT7) in thyroid cancer and positions them as a mechanistically promising and biomarker-stratifiable therapeutic hypothesis for the most clinically challenging contexts in the disease—BRAF inhibitor-resistant papillary thyroid cancer, RAI-refractory differentiated thyroid cancer, and anaplastic thyroid cancer. Three findings emerge as central to the integrated framework developed here. First, sirtuins exert isoform-specific dichotomous effects in thyroid cancer, with SIRT1, SIRT6, and SIRT7 acting as tumor promoters through the engagement of BRAF/MAPK, PI3K/AKT, EMT, Hippo, and post-translational modification axes, while SIRT3 and SIRT4 function as tumor suppressors through mitochondrial metabolic regulation. Second, recent recognition of non-canonical desuccinylation activity for SIRT7—with KIF23 in anaplastic thyroid cancer and LATS1 in papillary thyroid cancer as preclinically identified oncogenic substrates—fundamentally expands the mechanistic and therapeutic landscape, identifying an entirely new class of substrate-stratified targets. Third, the SIRT6–NCOA4 ferritinophagy axis in anaplastic thyroid cancer and the disease-state-specific dual role of SIRT6 in ferroptosis create a biologically rational framework for combining sirtuin modulation with ferroptosis-inducing agents in distinct subtype-specific settings.

These findings carry concrete implications for current treatment paradigms. For *BRAF* V600E-mutant disease (the population most directly impacted by the dabrafenib + trametinib combination), the SIRT6–BRAF/ERK/Mcl-1 axis provides a rational fourth-agent strategy with the potential to pre-empt MAPK reactivation, the principal resistance mechanism limiting current BRAF/MEK regimens. For RAI-refractory differentiated thyroid cancer (the largest unmet-need population currently treated with multikinase inhibitors), the SIRT6–HIF-1α–EMT axis provides a complementary anti-angiogenic/anti-EMT rationale, while the SIRT3/SIRT4 reactivation hypothesis offers a conceptually distinct route to redifferentiation and re-sensitization to radioiodine. For anaplastic thyroid cancer (the most lethal subtype, with no curative systemic therapy outside of *BRAF* V600E-mutant disease), SIRT6-stabilizing strategies combined with ferroptosis inducers (sulfasalazine, RSL3 derivatives) offer a mechanistically distinct vulnerability that should be prioritized for biomarker-driven preclinical validation. Across these contexts, the unifying clinical proposition is that sirtuin-targeted strategies are most likely to provide meaningful benefit when added to established backbones in biomarker-stratified populations, rather than evaluated as monotherapies in unselected patients.

Three principal challenges define the path from this mechanistic framework to clinical implementation. First, the isoform selectivity of currently available SIRT modulators remains imperfect, particularly for the substrate-stratified opportunities represented by SIRT7 desuccinylase inhibitors and for the activator/restoration approach required to harness tumor-suppressive SIRT3 and SIRT4. Second, validated predictive biomarkers for patient stratification are essentially absent—no current assay distinguishes thyroid cancer patients likely to benefit from SIRT-directed therapy, and the transcript-protein discordance documented in Section 3.5 (particularly for SIRT1 and SIRT7) indicates that protein-level rather than mRNA-level profiling will be required for clinically actionable assays. Third, no clinical trial of any SIRT modulator has been conducted specifically in thyroid cancer as of 2026, despite substantial preclinical rationale across multiple combinations—a gap that simultaneously represents the field’s principal limitation and its clearest opportunity for future translational research.

Taken together, the body of evidence reviewed here supports sirtuins as a genuinely promising target class for the most challenging contexts in thyroid cancer therapy, but one whose clinical implementation will require the coordinated development of isoform-selective compounds, validated predictive biomarkers, and disease-specific in vivo evidence. The structured therapeutic framework presented in this review—anchored by the three star-designated priority combinations (BRAF/MEK + SIRT6 inhibitor, multikinase inhibitor + SIRT6 inhibitor, ferroptosis inducer + SIRT6-stabilizing strategy)—is intended not as immediately actionable clinical practice but as a coherent translational hypothesis to guide preclinical investigation and biomarker-driven trial design over the next phase of research. The forward-looking priorities defining this next phase are presented in Section 10.

## 10. Future Directions

Building on the mechanistic and translational framework developed in this review, we identify five concrete research priorities that should guide the next phase of sirtuin-targeted research in thyroid cancer. These priorities are organized in order of immediate translational relevance and are designed to be actionable for laboratory researchers, translational scientists, and clinical investigators.

### 10.1. Biomarker Development for Patient Stratification

Predictive biomarker development should accompany—and ideally precede—drug discovery efforts. Specifically, the field requires (i) validated immunohistochemical assays for SIRT1, SIRT6, and SIRT7 protein expression in formalin-fixed paraffin-embedded thyroid tumor tissues, calibrated against orthogonal mass-spectrometry-based proteomic measurements to address the transcript-protein discordance documented in Section 3.5; (ii) substrate-specific post-translational modification assays for SIRT7 desuccinylation targets (LATS1 and KIF23), which would serve as direct pharmacodynamic markers for SIRT7-selective inhibitors; (iii) integrated multi-marker panels combining SIRT expression with mutational status (*BRAF* V600E, *PTEN*, *TP53*) and immune microenvironment markers (PD-L1, tumor-infiltrating lymphocytes, NAMPT); and (iv) clinical-grade assays compatible with the 2025 American Thyroid Association risk stratification framework, enabling SIRT-based stratification to complement rather than duplicate existing risk-adapted decision-making.

### 10.2. Isoform-Selective Drug Development

Three pharmacological gaps deserve focused attention. First, the development of SIRT7-selective desuccinylase inhibitors with thyroid cancer-relevant pharmacology represents a high-priority opportunity given the substrate-specific oncogenic axes (KIF23 in ATC; LATS1 in PTC) identified in 2024–2025 literature. Currently available SIRT7 modulators (97491 series and analogs) require further medicinal chemistry refinement to achieve drug-like properties suitable for in vivo evaluation. Second, the development of bona fide SIRT3 and SIRT4 activators (or alternative restoration strategies such as epigenetic agents or gene therapy approaches with thyroid-specific delivery vehicles) is required to translate the tumor-suppressive sirtuin paradigm into actionable therapeutics for redifferentiation strategies. Third, dual SIRT inhibitors (e.g., SIRT1 + SIRT6) with optimized pharmacokinetics and on-target/off-target selectivity should be explored for the parallel-pathway resistance settings that single-isoform inhibition is unlikely to fully address.

### 10.3. Biomarker-Driven Preclinical Validation of Combination Strategies

The fourteen combinations summarized in Table 4 require rigorous biomarker-driven preclinical validation before clinical translation. The three star-designated priority combinations should be advanced first: (i) OSS_128167 + dabrafenib/trametinib in *BRAF* V600E-mutant PTC and ATC patient-derived xenografts and organoids, with MAPK reactivation kinetics as the primary pharmacodynamic endpoint; (ii) OSS_128167 + lenvatinib or sorafenib in RAI-refractory DTC models, with HIF-1α/EMT axis modulation as the mechanistic readout and tumor microvascular density as the histological correlate; and (iii) SIRT6-stabilizing strategies + sulfasalazine in SIRT6-high ATC xenografts, with intratumoral iron quantification, lipid peroxidation, and NCOA4-dependent ferritin degradation as ferroptosis-axis endpoints. Subsequent priorities include SIRT2 inhibitor + anti-PD-1 immune checkpoint inhibitor combinations in immunocompetent ATC models with assessment of tumor-reactive CD8^+^ T-cell metabolic fitness, and SIRT3 activator + MEK inhibitor + RAI re-sensitization in dedifferentiated thyroid cancer models with NIS restoration as the primary endpoint.

### 10.4. Mechanistic Studies of Underexplored Sirtuins

Two sirtuins remain mechanistically underexplored in thyroid cancer and represent important hypothesis-generating opportunities. First, the pronounced SIRT5 downregulation observed in ATC (Section 3.2) suggests that ATC may exhibit a coordinated collapse of multiple mitochondrial sirtuins extending beyond the canonical axes. Dedicated SIRT5-focused studies investigating substrate-level succinylation, malonylation, and glutarylation profiles of TCA cycle and electron transport chain enzymes (CPS1, succinate dehydrogenase, acyl-CoA dehydrogenases) in ATC models, together with functional rescue experiments using SIRT5 reconstitution, would define whether SIRT5 loss contributes causally to the metabolic dysfunction characteristic of ATC. Second, SIRT2-focused investigations in the thyroid cancer immune microenvironment are warranted, given the emerging recognition of SIRT2 as a T-cell metabolic checkpoint (Section 5.3); systematic profiling of SIRT2 expression in tumor-infiltrating lymphocytes and assessment of AGK2 or AK-7 pharmacology in immunocompetent thyroid cancer models would clarify whether SIRT2 inhibition can be advanced as a clinical immune-axis strategy.

### 10.5. Toward First-in-Disease Clinical Evaluation: Preclinical Prerequisites

Clinical translation remains the ultimate goal, but it is explicitly contingent on the successful preclinical validation described in Section 10.3. No SIRT-targeted clinical trial has yet been conducted in thyroid cancer, and no proposed combination currently satisfies all three prerequisites for clinical entry: thyroid cancer-specific in vivo efficacy, demonstrated pharmacological feasibility, and an established safety rationale. Once these prerequisites are met for one or more of the star-designated combinations, a thyroid cancer-specific basket trial could be considered, with three parallel biomarker-defined cohorts mirroring the three priority combinations: (cohort 1) *BRAF* V600E-mutant PTC or ATC receiving a SIRT6 inhibitor added to dabrafenib + trametinib; (cohort 2) RAI-refractory DTC receiving a SIRT6 inhibitor added to lenvatinib; and (cohort 3) SIRT6-high ATC receiving a SIRT6-stabilizing agent plus sulfasalazine, with mandatory pre- and on-treatment biopsies for pharmacodynamic readouts (MAPK reactivation, HIF-1α expression, and lipid peroxidation, respectively). We present this design as an illustrative framework for how such trials could eventually be structured, not as a near-term recommendation. Achieving the five priorities outlined above would convert the present framework from a structured therapeutic hypothesis into an evidence-based clinical strategy; until then, it should guide preclinical investigation and biomarker-driven trial design rather than clinical practice.

## Figures and Tables

**Table 1:** Comprehensive summary of sirtuin family members and their roles in thyroid cancer.

SIRT	Localization	Enzymatic Activity	Expression in TC	Role in TC	Key Substrates/Pathways	Subtype	Clinical Implication	Refs.
SIRT1	Nucleus, cytoplasm	Deacetylase	Upregulated	Tumor promoter	PTEN, p53, FOXO; PI3K/AKT axis	PTEN-deficient PTC, FTC	Promotes tumorigenesis; chemoresistance to etoposide	[23,73]
SIRT2	Cytoplasm, nucleus	Deacetylase	Downregulated in DTC	Context-dependent (immune)	Tubulin, mitotic regulators	All subtypes (immune compartment); DTC (tumor cell)	Limited tumor-cell-intrinsic data; emerging T-cell metabolic checkpoint relevant to ICI combinations	[30]
SIRT3	Mitochondria	Deacetylase	Downregulated in DTC	Tumor suppressor	SOD2, IDH2, OXPHOS regulators	DTC	Loss promotes ROS accumulation and metabolic reprogramming	[29,30]
SIRT4	Mitochondria	ADP-ribosyl-T, lipoamidase	Downregulated	Tumor suppressor	GDH, glutamine metabolism	PTC	Inhibits proliferation and invasion via glutamine metabolism blockade	[74]
SIRT5	Mitochondria	Desuccinylase, demalonylase, deglutarylase	Downregulated in DTC	Underexplored	Metabolic enzymes (CPS1, SDH)	Limited TC data	Potential metabolic regulator; clinical relevance unclear	[30,37]
SIRT6	Nucleus	Deacetylase, ADP-ribosyl-T	Upregulated	Tumor promoter	BRAF/ERK/Mcl-1, HIF-1α	PTC (aggressive), ATC	Promotes EMT, invasion, *BRAF* V600E-driven aggressiveness; ferroptosis regulator	[26,28,33]
SIRT7	Nucleus (nucleolus)	Deacetylase, desuccinylase	Upregulated	Tumor promoter (context-dependent in immune compartment)	AKT/p70S6K1 via DBC1/SIRT1; KIF23, LATS1 (desuccinylation)	PTC, ATC	Promotes proliferation and migration via non-canonical PTM regulation	[24,25,27]

The seven mammalian sirtuins (SIRT1–7) display distinct subcellular localizations, enzymatic activities, and expression patterns in thyroid cancer (TC), with predominantly tumor-promoting or tumor-suppressive roles in reported TC models, recognizing that these classifications are context-dependent and may vary by histological subtype, genetic background, and disease stage. SIRT1, SIRT6, and SIRT7 predominantly function as tumor promoters in available TC evidence, particularly in differentiated and anaplastic thyroid cancer, by activating PI3K/AKT, BRAF/ERK, and HIF-1α pathways, whereas SIRT3 and SIRT4 are typically downregulated and predominantly act as tumor suppressors in available TC evidence through mitochondrial metabolic regulation. SIRT7 is emerging as a desuccinylase that modifies KIF23 and LATS1 to promote proliferation and migration in anaplastic and papillary thyroid cancer, respectively. SIRT2 and SIRT5 remain underexplored in thyroid cancer. Expression in TC refers to protein-level status where reported; mRNA–protein discordance, notably for SIRT1, is discussed in Section 3.5. ATC, anaplastic thyroid cancer; CPS1, carbamoyl phosphate synthetase 1; DBC1, deleted in breast cancer 1; DTC, differentiated thyroid cancer; EMT, epithelial–mesenchymal transition; ERK, extracellular signal-regulated kinase; FOXO, forkhead box O; FTC, follicular thyroid cancer; GDH, glutamate dehydrogenase; HIF-1α, hypoxia-inducible factor 1-alpha; IDH2, isocitrate dehydrogenase 2; KIF23, kinesin family member 23; LATS1, large tumor suppressor 1; Mcl-1, myeloid cell leukemia 1; OXPHOS, oxidative phosphorylation; p70S6K1, p70 S6 kinase 1; PI3K, phosphoinositide 3-kinase; PTC, papillary thyroid cancer; PTEN, phosphatase and tensin homolog; PTM, post-translational modification; ROS, reactive oxygen species; SDH, succinate dehydrogenase; SOD2, superoxide dismutase 2; TC, thyroid cancer.

**Table 2:** Evidence levels for the principal SIRT–thyroid cancer mechanistic axes discussed in this review.

Axis	Evidence Type	Thyroid-Specific?	In Vivo?	Clinical Correlation	Therapeutic Readiness
SIRT6–BRAF/ERK/Mcl-1	Cell + xenograft	Yes (PTC)	Partial	Limited	Preclinical
SIRT4–GDH/EMT suppression	205-PTC IHC + GEO/TCGA + 3 cell lines + xenograft	Yes (PTC)	Yes (B-CPAP xenograft)	Moderate (n = 205; ETE *p* < 0.001; OS *p* = 0.016)	Preclinical (gene therapy proof-of-concept)
SIRT7–KIF23 desuccinylation	Cell-based	Yes (ATC)	Limited	Limited	Early preclinical
SIRT7–LATS1 desuccinylation	Cell + xenograft (single lab, replication needed)	Yes (PTC)	Yes	Limited	Preclinical
SIRT6–NCOA4 ferroptosis	Preclinical	Yes (ATC)	Needs clarification	None/limited	Hypothesis-generating
NAMPT–SIRT1–PD-L1	Mixed cancer evidence	Partial	Limited	Limited	Hypothesis-generating

**Table 3:** SIRT modulators with relevance to thyroid cancer—comprehensive summary.

Compound	Type	Primary Target	Selectivity	Chemical Class	Mechanism	TC Preclinical Evidence	Highest Clinical Phase	Refs.
Resveratrol	Activator	SIRT1	Pan-target (multi)	Stilbene polyphenol	Allosteric activation, ↓ Km for substrates	Anti-proliferative in PTC lines (B-CPAP, K1, TPC-1); accelerated tumors in *Pten*-null mice	Phase II/III (multi-cancers, not TC)	[23,153]
SRT1720	Activator	SIRT1	SIRT1-selective (~3 µM)	Imidazo-thiazole	Allosteric activation	Accelerated thyroid tumorigenesis in *Pten*-null mice	Discontinued (preclinical limits)	[23]
SRT2104	Activator	SIRT1	SIRT1-selective	Synthetic	Allosteric activation	None reported in TC	Phase I/II (metabolic, inflammatory)	[154,155]
SRT2379	Activator	SIRT1	SIRT1-selective	Synthetic	Allosteric activation	None in TC	Phase I (metabolic disease)	[156]
Honokiol	Activator	SIRT3	Multi-target	Biphenolic natural product	Direct SIRT3 binding/activation	Limited TC data; preclinical antitumor in other cancers	Preclinical; supplement use	[157,158]
MDL-800	Activator	SIRT6	SIRT6-selective	Quinazoline	Allosteric activation	None in TC	Preclinical	[37,159]
MDL-801	Activator	SIRT6	SIRT6-selective (improved)	Quinazoline	Allosteric activation	None in TC	Preclinical	[37]
EX-527 (Selisistat)	Inhibitor	SIRT1	SIRT1-selective; >200× vs. SIRT2/3; sub-100 nM IC50 range	Indole carboxamide	Competitive (NAD^+^/substrate trap)	Reverses chemoresistance to etoposide in TC cells	Phase II (Huntington’s; NCT04184323)	[73,160]
Sirtinol	Inhibitor	SIRT1/SIRT2	Non-selective	β-naphthol-sulfonamide	Competitive	Tool compound in TC studies; superseded	Preclinical	[161]
Cambinol	Inhibitor	SIRT1/SIRT2	Dual SIRT1/2	β-naphthol	Competitive	None in TC; activity in Burkitt lymphoma	Preclinical	[162]
Inauhzin	Inhibitor	SIRT1	SIRT1-selective	Indolyl-azine	p53 reactivation (indirect)	None in TC	Preclinical	[163]
AGK2	Inhibitor	SIRT2	SIRT2-selective	Cyano-styrylcarboxamide	Competitive	None in TC; brain-permeable	Preclinical (CNS focus)	[164]
AK-7	Inhibitor	SIRT2	SIRT2-selective (improved PK)	Optimized AGK2 derivative	Competitive	None in TC	Preclinical	[165]
3-TYP	Inhibitor	SIRT3	SIRT3-selective	Tetrazolylphenyl pyridine	Competitive	Tool compound (caution: SIRT3 is suppressor in TC)	Preclinical	[166]
OSS_128167	Inhibitor	SIRT6	SIRT6-selective	Pyrrolopyrimidine	Competitive	Strong rationale for TC: BRAF/ERK and HIF-1α/EMT axes; preclinical activity in MM, PDAC	Preclinical	[26,28,104]
97491/imidazothiazoles	Inhibitor	SIRT7	SIRT7-selective (early)	Imidazothiazole	Competitive (low µM IC50)	None in TC; emerging priority for ATC and PTC given KIF23/LATS1 axes	Preclinical (early)	[24,25,27]
Nicotinamide	Inhibitor	Pan-SIRT	None (endogenous)	Vitamin B3 amide	End-product feedback inhibition	Used in non-melanoma skin cancer chemoprevention	Phase III (other)	[167]
MC2494	Inhibitor	Pan-SIRT	Non-selective	Synthetic	Competitive	Anti-proliferative in multiple cancer lines (not TC-specific)	Preclinical	[168]

Sirtuin-modulating compounds are organized into activators (STACs; rows 1–7) and inhibitors (STICs; rows 8–18), with primary targets, selectivity profiles, chemical classes, mechanisms of action, available preclinical evidence in thyroid cancer (TC), and the highest clinical trial phase reached. Notably, no compound has been evaluated in a clinical trial specifically for thyroid cancer as of 2025, despite substantial preclinical evidence supporting several candidates (EX-527 for SIRT1, OSS_128167 for SIRT6) and the recent emergence of SIRT7 desuccinylase inhibitors as a priority area for anaplastic and papillary thyroid cancer. ATC, anaplastic thyroid cancer; MM, multiple myeloma; PDAC, pancreatic ductal adenocarcinoma. CNS, central nervous system; ERK, extracellular signal-regulated kinase; HIF-1α, hypoxia-inducible factor 1-alpha; IC50, half-maximal inhibitory concentration; Km, Michaelis constant; KIF23, kinesin family member 23; LATS1, large tumor suppressor 1; PK, pharmacokinetics; PTC, papillary thyroid cancer; SIRT, sirtuin; STAC, sirtuin-activating compound; STIC, sirtuin inhibitor compound; TC, thyroid cancer.

**Table 4:** Combination strategies for SIRT modulators in thyroid cancer—clinical translation framework.

#	SIRT Modulator	Combination Partner(s)	Therapeutic Class	Mechanistic Rationale	Target Population	Predictive Biomarker(s)	Evidence Level	Translation Readiness
1	OSS_128167 (SIRT6 inhibitor)	Dabrafenib + trametinib	BRAF/MEK inhibitor combination	SIRT6 amplifies BRAF/ERK via Mcl-1; pre-empts MAPK reactivation	*BRAF* V600E PTC, ATC	*BRAF* V600E, SIRT6 expression	Strong preclinical	Preclinical
2	EX-527 (SIRT1) + OSS_128167 (SIRT6)	Dabrafenib + trametinib + CB-839	BRAFi + MEKi + glutaminase inhibitor	Dual SIRT inhibition closes PI3K/AKT and glutamine escape routes	*BRAF* V600E ATC	*BRAF* V600E, PTEN, glutamine dependency	Mechanistic only	Conceptual
3	OSS_128167 (SIRT6 inhibitor)	Lenvatinib or sorafenib	Multikinase inhibitor	SIRT6 inhibition attenuates HIF-1α/EMT and angiogenesis	RAI-refractory DTC	SIRT6 expression, HIF-1α, EMT score	Strong preclinical	Preclinical
4	EX-527 (SIRT1 inhibitor)	Lenvatinib or cabozantinib	Multikinase inhibitor	SIRT1 inhibition disrupts PI3K/AKT axis driving acquired VEGFR resistance	RAI-refractory DTC after MKI failure	SIRT1 expression, PTEN status	Mechanistic	Preclinical
5	SIRT modulator (e.g., OSS_128167)	Anlotinib	MKI with intrinsic ferroptosis activity	Anlotinib induces ferroptosis; SIRT modulation potentiates	RAI-refractory DTC, ATC	SIRT6, GPX4, NCOA4	Preliminary preclinical	Preclinical
6	EX-527 or OSS_128167	Pembrolizumab or spartalizumab	Anti-PD-1 ICI	Disrupts NAMPT-SIRT1-PD-L1 axis and SIRT6 Treg functions	PD-L1-low/immune-cold ATC, advanced PTC	PD-L1, NAMPT, Treg infiltration, TMB	Mechanistic; cross-tumor	Preclinical
7	SIRT2 inhibitor (AGK2, AK-7)	Pembrolizumab or nivolumab	Anti-PD-1 ICI	SIRT2 inhibition enhances tumor-reactive CD8+ T-cell metabolic fitness and effector function	*BRAF* V600E PTC, ATC	CD8+ T cell infiltration, SIRT2	Cross-tumor preclinical	Preclinical (tool inhibitors available)
8	SIRT7-selective inhibitor (97491 series)	Anti-PD-1 ICI	Anti-PD-1 ICI	Targets tumor-intrinsic SIRT7-KIF23/LATS1; modulates PD-L1	ATC (KIF23), aggressive PTC (LATS1)	SIRT7, KIF23/LATS1 succinylation	Early preclinical	Inhibitor dev needed
9	SIRT6-stabilizing strategies	Sulfasalazine	System Xc^−^ inhibitor (ferroptosis)	NCOA4-mediated ferritinophagy synergizes with cystine/glutamate antiporter blockade	SIRT6-overexpressing ATC	SIRT6 high, NCOA4	Strong preclinical	Preclinical priority
10	SIRT modulator (e.g., OSS_128167)	RSL3 derivatives or GPX4-targeting agents	GPX4 inhibitor	GPX4 blockade in SLC7A11-amplified tumors; SIRT context modulates	ATC with SLC7A11 amplification	SLC7A11, GPX4, lipid peroxidation	Preclinical	Preclinical
11	SIRT modulator (e.g., OSS_128167)	Vitamin C, neferine, curcumin, or shikonin	Natural compound ferroptosis inducer	Low-toxicity ferroptosis induction in elderly/comorbid patients	Elderly ATC, comorbid DTC	SIRT6, ACSL4, lipid peroxidation	Preclinical (natural side)	Phase I feasibility
12	OSS_128167 (SIRT6 inhibitor)	Dabrafenib + trametinib + GPX4 blockade	BRAFi + MEKi + ferroptosis (triplet)	Triplet integrating MAPK blockade, ferroptosis induction, SIRT6 axis	*BRAF* V600E ATC	*BRAF* V600E, GPX4, SIRT6	Triplet validated (without SIRT6); addition logical	Preclinical priority
13	SIRT3 activator (e.g., honokiol)	Selumetinib + RAI	MEKi + RAI re-sensitization	SIRT3 reverses metabolic dedifferentiation; MEK restores NIS; RAI provides cytotoxicity	RAI-refractory DTC	NIS, SIRT3/SIRT4, BRAF	Conceptual	POC trial design
14	Epigenetic agent restoring SIRT3/SIRT4	RAI re-administration	Epigenetic + RAI	Restoration of suppressor SIRT expression reverses dedifferentiation	Highly dedifferentiated DTC	DNA methylation of SIRT3/4 promoters	Preliminary	Conceptual

Fourteen combination strategies are presented, organized by therapeutic class (BRAF/MEK inhibitors, multikinase inhibitors, immune checkpoint inhibitors, ferroptosis inducers, and radioiodine re-sensitization regimens) following the structure of Section 8. Each entry specifies the SIRT modulator, combination partner(s), mechanistic rationale, target patient population, predictive biomarkers required for patient selection, evidence level, and developmental status. Three combinations (rows 1, 3, and 9, marked with bold borders) are designated as having the highest priority for biomarker-driven preclinical validation. ACSL4, acyl-CoA synthetase long-chain family member 4; ATC, anaplastic thyroid cancer; BRAF, v-Raf murine sarcoma viral oncogene homolog B; BRAFi, BRAF inhibitor; CB-839, telaglenastat (glutaminase inhibitor); DTC, differentiated thyroid cancer; EMT, epithelial–mesenchymal transition; ERK, extracellular signal-regulated kinase; GPX4, glutathione peroxidase 4; HIF-1α, hypoxia-inducible factor 1-alpha; ICI, immune checkpoint inhibitor; KIF23, kinesin family member 23; LATS1, large tumor suppressor 1; MEKi, MEK inhibitor; MKI, multikinase inhibitor; NAMPT, nicotinamide phosphoribosyltransferase; NCOA4, nuclear receptor coactivator 4; NIS, sodium-iodide symporter; PD-1, programmed cell death 1; PD-L1, programmed death-ligand 1; PI3K, phosphoinositide 3-kinase; POC, proof of concept; PTC, papillary thyroid cancer; PTEN, phosphatase and tensin homolog; RAI, radioactive iodine; SIRT, sirtuin; SLC7A11, solute carrier family 7 member 11; TMB, tumor mutational burden; VEGFR, vascular endothelial growth factor receptor.

## Data Availability

No new primary data were generated in this study. Figure 2 was produced by the authors through secondary analysis of publicly available data retrieved from open-access databases; the original datasets are freely accessible from their respective repositories under their stated terms of use, and the corresponding source publications are cited in the figure legend and in the reference list. The analytical workflow, processed datasets, and additional details underlying Figure 2 are provided in the Supplementary Methods and Supplementary Data accompanying this article. All other data discussed in this article are from previously published sources cited in the references.

## Supplementary Materials

The following supporting information can be downloaded at: https://www.mdpi.com/article/10.3390/cancers18132093/s1, Supplementary Methods: Detailed methodology for the integrative transcriptomic analysis presented in Figure 2, including dataset overview, cohort selection, data acquisition and preprocessing, z-score normalization, statistical analysis, software, and limitations of the integrative approach (Sections S1.1–S1.7; Table S1). Supplementary Data: Complete per-sample SIRT1–SIRT7 z-score matrix and corresponding expression values for all cohorts used in the Figure 2 analysis.

## Abbreviations

The following abbreviations are used in this manuscript:

ATC	anaplastic thyroid cancer
DTC	differentiated thyroid cancer
EMT	epithelial–mesenchymal transition
FTC	follicular thyroid cancer
GDH	glutamate dehydrogenase
GPX4	glutathione peroxidase 4
ICI	immune checkpoint inhibitor
KIF23	kinesin family member 23
LATS1	large tumor suppressor kinase 1
MAPK	mitogen-activated protein kinase
MDSC	myeloid-derived suppressor cell
MTC	medullary thyroid cancer
NAD^+^	nicotinamide adenine dinucleotide
NAMPT	nicotinamide phosphoribosyltransferase
NIS	sodium–iodide symporter
OXPHOS	oxidative phosphorylation
PD-L1	programmed death ligand 1
PI3K	phosphoinositide 3-kinase
PROTAC	proteolysis-targeting chimera
PTC	papillary thyroid cancer
RAI	radioactive iodine
ROS	reactive oxygen species
SDH	succinate dehydrogenase
SIRT	sirtuin
SLC7A11	solute carrier family 7 member 11
SOD2	superoxide dismutase 2
STAC	sirtuin-activating compound
STIC	sirtuin inhibitor compound
TAM	tumor-associated macrophages
TC	thyroid cancer
TLS	tertiary lymphoid structure
TME	tumor microenvironment
Treg	regulatory T cell
YAP/TAZ	yes-associated protein/transcriptional coactivator with PDZ-binding motif
