# Supplementary material for: Targeting Sirtuins in Thyroid Cancer: Mechanisms, Drug Development, and Emerging Roles in Tumor Immunity and Ferroptosis
Source: Cancers (Basel). 2026 Jun 27;18(13):2093. doi: 10.3390/cancers18132093 (PMC13359528; doi:10.3390/cancers18132093)
Supplement: Supplementary file 1 [file cancers-18-02093-s001.zip › Supplementary Methods_R2.pdf]

# Supplementary Methods (Figure 2)

### *S1.1. Overview*

Figure 2 integrates publicly available transcriptomic data from one large RNA-sequencing cohort (TCGA-THCA) and six independent microarray cohorts (GSE27155, GSE29265, GSE33630, GSE65144, GSE196264, GSE197861) to provide a subtype-stratified view of SIRT1–SIRT7 mRNA expression across the major thyroid cancer histological subtypes (PTC, FTC, MTC, ATC) and matched normal thyroid tissue. This integrative analysis is presented as a conceptual summary of currently available transcriptomic evidence rather than as a single, uniformly normalized, cross-platform meta-analysis, and the inferences drawn from it are presented as directional rather than strictly quantitatively comparable across cohorts.

### *S1.2. Cohort Selection*

Cohorts were selected based on the following criteria: (i) public availability with full sample-level annotation, (ii) inclusion of both tumor and reference (non-tumor) thyroid samples (for normalization reference), (iii) sufficient sample size ($n \geq 3$ for rare subtypes; $n \geq 20$ for PTC), (iv) availability of a probe or transcript measurement for at least 6 of the 7 SIRT genes, and (v) annotation of histological subtype consistent with the WHO/ATA classification. The TCGA-THCA cohort was selected for PTC due to its substantially larger sample size compared to all microarray PTC cohorts. Microarray cohorts (GSE27155, GSE29265, GSE33630, GSE65144, GSE196264, GSE197861) were retained for the rare subtypes (FTC, MTC, ATC) because no comparable large RNA-seq cohort exists in the literature for these subtypes.

We acknowledge two important limitations: (a) the TCGA-THCA cohort predominantly represents classical and follicular variant PTC and does not capture the full molecular spectrum of FTC, MTC, or ATC; and (b) microarray cohorts differ in profiling platform, probe coverage, and normalization, which we address by within-cohort z-score normalization (Section S1.4) rather than inter-platform batch correction.

### *S1.3. Data Acquisition and Preprocessing*

TCGA-THCA gene-level transcript counts and clinical annotations were obtained from the Genomic Data Commons (GDC) data portal (https://portal.gdc.cancer.gov/). STAR-aligned, TPM-normalized expression values were used in log2 (TPM+1) form for z-score computation. Of 564 thyroid samples (excluding metastatic thyroid cancer) available in TCGA-THCA, 505 PTC tumor samples and 59 matched normal thyroid samples (the subset with both expression data and confirmed pathological annotation) were retained for analysis.

Microarray datasets (GSE27155, GSE29265, GSE33630, GSE65144, GSE196264, GSE197861) were downloaded from the NCBI Gene Expression Omnibus (https://www.ncbi.nlm.nih.gov/geo/) using the GEOparse Python package. Probe-level expression matrices were processed using each dataset's deposited normalization method (RMA for Affymetrix HG-U133A/HG-U133 Plus 2.0; Illumina BeadStudio quantile normalization for Illumina HumanHT-12 WG-DASL). Probes were mapped to gene symbols using the corresponding platform annotation files; when multiple probes mapped to a single SIRT

gene, expression values were averaged across all matching probes.

### *S1.4. Z-Score Normalization*

Because the seven cohorts span both RNA-seq and microarray platforms with substantially different dynamic range and noise characteristics, expression values were converted to dataset-internal z-scores using each cohort's reference (non-tumor) samples as the reference distribution. The reference samples were histologically normal thyroid tissue in all cohorts except GSE197861, in which benign nodular goiter served as the reference. Specifically, for each gene g in dataset d, $z(s,g) = (x(s,g) - \mu(ref,g,d)) / \sigma(ref,g,d)$, where $\mu(ref,g,d)$ and $\sigma(ref,g,d)$ are the mean and standard deviation of expression of gene g across the reference samples in cohort d. This within-cohort normalization removes the bulk of platform-specific scaling and inter-cohort batch effects without requiring explicit inter-platform batch correction (e.g., ComBat), which could risk overcorrecting biologically meaningful subtype-specific signals in rare-subtype cohorts. Z-scores were clipped to [−3, +3] for heatmap visualization purposes only; statistical testing was performed on uncapped values.

### *S1.5. Statistical Analysis*

Subtype-vs-normal contrasts were tested using the two-sided Wilcoxon rank-sum (Mann-Whitney U) test on per-sample z-scores, with the reference defined as the cohort-matched reference samples. P-values were adjusted for multiple testing across all SIRT × subtype contrasts (7 genes × 4 subtypes = 28 tests) using the Benjamini-Hochberg (BH) false discovery rate procedure. Significance thresholds shown in Figure 2 are: $*q < 0.05$, $**q < 0.01$, $***q < 0.001$. Hierarchical clustering of samples within each subtype was performed using Ward's linkage on Euclidean distance computed across the SIRT1–SIRT7 z-score vectors.

### *S1.6. Software*

All analyses were performed in Python 3.14.5 using pandas, numpy, scipy.stats (Wilcoxon rank-sum test), statsmodels (BH-FDR correction), matplotlib (visualization), and scipy.cluster.hierarchy (Ward linkage). The complete per-sample z-score matrix (Figure2_z-score data.csv) is provided as Supplementary Data File 1.

### *S1.7. Limitations of the Integrative Approach*

Three caveats are emphasized: (i) inter-platform comparison limitation — TCGA-THCA is RNA-seq while FTC/MTC/ATC cohorts are microarray, so absolute fold-change comparisons between PTC and other subtypes should be interpreted directionally rather than quantitatively; (ii) sample size disparity — PTC (n = 625) statistical power vastly exceeds that of rare subtypes (FTC n = 17, MTC n = 19, ATC n = 36), meaning that null findings in rare subtypes do not exclude meaningful biological differences but instead reflect underpowered detection; (iii) probe coverage gap — SIRT7 is not represented on the Illumina HumanHT-12 WG-DASL platform used for GSE196264 (MTC cohort), and the corresponding MTC × SIRT7 cell is shown as N/A in Figure 2 rather than imputed. (iv) reference-tissue heterogeneity — the

reference (non-tumor) group was histologically normal thyroid tissue in all cohorts except GSE197861, in which benign nodular goiter was used; because z-scores were computed within each cohort against its own reference and combined only directionally, this does not affect the qualitative conclusions but is noted here as a limitation. These limitations are why Figure 2 is presented as a conceptual integrative summary rather than a definitive meta-analysis, and why both immunohistochemical/Western validation (for SIRT1; see §3.1) and dedicated proteomic studies in rare subtypes (for SIRT5, SIRT7; see §9) remain priorities for the field.

### *Table S1. Cohort Information, Platforms, and Sample Sizes for Figure 2*

| Cohort | Subtype | n | Platform | Data type | Normalization | SIRT7 probe |
|---|---|---|---|---|---|---|
| TCGA-THCA | Normal | **59** | Illumina HiSeq RNA-seq | RNA-seq (STAR + RSEM) | log2(TPM+1) | Present |
| TCGA-THCA | PTC | **505** | Illumina HiSeq RNA-seq | RNA-seq (STAR + RSEM) | log2(TPM+1) | Present |
| GSE27155 | Normal | **4** | Affymetrix HG-U133A (GPL96) | Microarray | log10 → log2 (×3.32) | Present |
| GSE27155 | PTC | **51** | Affymetrix HG-U133A (GPL96) | Microarray | log10 → log2 (×3.32) | Present |
| GSE27155 | FTC | **13** | Affymetrix HG-U133A (GPL96) | Microarray | log10 → log2 (×3.32) | Present |
| GSE27155 | ATC | **4** | Affymetrix HG-U133A (GPL96) | Microarray | log10 → log2 (×3.32) | Present |
| GSE27155 | MTC | **2** | Affymetrix HG-U133A (GPL96) | Microarray | log10 → log2 (×3.32) | Present |
| GSE29265 | Normal | **20** | Affymetrix HG-U133 Plus 2.0 (GPL570) | Microarray | log2 RMA | Present |
| GSE29265 | PTC | **20** | Affymetrix HG-U133 Plus 2.0 (GPL570) | Microarray | log2 RMA | Present |
| GSE29265 | ATC | **9** | Affymetrix HG-U133 Plus 2.0 (GPL570) | Microarray | log2 RMA | Present |
| GSE33630 | Normal | **45** | Affymetrix HG-U133 Plus 2.0 (GPL570) | Microarray | log2 RMA | Present |
| GSE33630 | PTC | **49** | Affymetrix HG-U133 Plus 2.0 (GPL570) | Microarray | log2 RMA | Present |
| GSE33630 | ATC | **11** | Affymetrix HG-U133 Plus 2.0 (GPL570) | Microarray | log2 RMA | Present |
| GSE65144 | Normal | **13** | Affymetrix HG-U133 Plus 2.0 (GPL570) | Microarray | log2(x+1) applied | Present |
| GSE65144 | ATC | **12** | Affymetrix HG-U133 Plus 2.0 (GPL570) | Microarray | log2(x+1) applied | Present |
| GSE196264 | Normal | **3** | Illumina HumanHT-12 WG-DASL (GPL18281) | Microarray (DASL) | log2 + robust spline | Absent (N/A) |
| GSE196264 | MTC | **17** | Illumina HumanHT-12 WG-DASL (GPL18281) | Microarray (DASL) | log2 + robust spline | Absent (N/A) |
| GSE197861 | Nodular goiter | **6** | Affymetrix Clariom S (GPL23159) | Microarray | log2 | Present |
| GSE197861 | FTC | **4** | Affymetrix Clariom S (GPL23159) | Microarray | log2 | Present |

| Cohort | Subtype | n | Platform | Data type | Normalization | SIRT7 probe |
|---|---|---|---|---|---|---|
| **TOTAL** | **All subtypes** | **847** | *Mixed (1 RNA-seq + 5 microarray platforms)* | *Mixed* | *z-score (within-cohort)* | — |

**Notes:** *n indicates the number of samples retained after quality filtering. Total cancer samples: PTC 625 + FTC 17 + MTC 19 + ATC 36 = 697. Total normal samples: 150 (TCGA-THCA 59 + GSE27155 4 + GSE29265 20 + GSE33630 45 + GSE65144 13 + GSE196264 3 + GSE197861 6). Total samples in Figure 2 analysis: 847. SIRT7 absence on GPL18281 (GSE196264) is the reason the MTC × SIRT7 cell in Figure 2A is shown as N/A.*
